# Supplementary material for: Oral processing behavior and dental caries; an insight into a new relationship
Source: PLoS One. 2024 Jul 2;19(7):e0306143. doi: 10.1371/journal.pone.0306143 (PMC11218957; doi:10.1371/journal.pone.0306143)
Supplement: S1 File — (PDF) [file pone.0306143.s001.pdf]

Answer Key (Text-Based Questions 1-20)

From texture point of view, I prefer 1(certain texture) over 2: (different texture) as in Cattaneo et al	من حيث القوام افضل 1 (ذو قوام معين) مقارنة ب 2 (ذو قوام اخر) كما في الورقة العلمية من Cattaneo et al

**Answer**	**Meaning**	**المعنى**	**الاجابة**
Strongly agree	Prefer 1 only, would never go for 2	أفضّل فقط ١ ، لا أفضّل ٢ ابداً	اوافق بشدة
Agree	Always prefer 1, but might go for 2 once in a while	أفضّل ١ دائماً، لكن قد أفضّل ٢ في بعض الأحيان	اوافق
Somewhat agree	Like both but inclined towards 1	أفضّل كلاهما، لكن أميل نحو ١	اوافق نوعا ما
Somewhat disagree	Like both but inclined towards 2	أفضّل كلاهما، لكن أميل نحو ٢	لا اوافق نوعا ما
Disagree	Prefer 2 but might go for 1 once in a while	أفضّل ٢ دائماً، لكن قد أفضّل ١ في بعض الأحيان	لا اوافق
Strongly disagree	Prefer 2 only, would never go for 1	أفضّل فقط ٢، لا أفضّل ١ ابداً	لا اوافق بشدة

Answer Key (Photo-Based Questions 21-24)

Please look at the picture and state to what extent you agree with the following statement: I find great joy and pleasure in consuming products like these that I can (--- as in Cattaneo et al.	الرجاء النظر الى الصور التالية و اعلامنا الى اي مدى توافق على الجملة الاتية: استمتع في تناول هذه المجموعة من الاطعمة التي ------ -- كما في الورقة العلمية من Cattaneo et al

**Answer**	**Meaning**	**المعنى**	**الاجابة**
Strongly agree	I always enjoy this group of food	دائما استمتع بهذه المجموعة من الاطعمة	اوافق بشدة
Agree	Most of the time, I enjoy this group of food	معظم الوقت استمتع بهذه المجموعة من الاطعمة	اوافق
Somewhat agree	Frequently, I enjoy this group of food	مرارا استمتع بهذه المجموعة من الاطعمة	اوافق نوعا ما
Somewhat disagree	Occasionally, I enjoy this group of food	من حين لاخر استمتع بهذه المجموعة من الاطعمة	لا اوافق نوعا ما
Disagree	Rarely, I enjoy this group of food	نادرا ما استمتع بهذه المجموعة من الاطعمة	لا اوافق
Strongly disagree	I never enjoy this group of food	لا استمتع بهذه المجموعة من الاطعمة	لا اوافق بشدة
