# Supplementary material for: Oral processing behavior and dental caries; an insight into a new relationship
Source: PLoS One. 2024 Jul 2;19(7):e0306143. doi: 10.1371/journal.pone.0306143 (PMC11218957; doi:10.1371/journal.pone.0306143)
Supplement: S2 File — (PDF) [file pone.0306143.s002.pdf]

Preferred Oral Processing Behaviour (POPB) Questionnaire*

Question Number	English Version	Arabic Version
Q1	"I usually prefer a chewy piece of candy like e.g., wine gum over a hard piece of candy"	1- من حيث القوام, عادة ما افضل قطعة حلوى مطاطية (جيلاتينية يمكن مضغها) اكثر من قطعة حلوى صلبة (ملبس يمكن مصه)
Q2	"I usually prefer chocolate with crunchy fillings like nuts over chocolate that easily melts in my mouth"	2- من حيث القوام, عادة ما افضل اكل قطعة شوكولاته محشوة بالمكسرات اكثر من الشوكولاته السادة التي تذوب بسهولة في الفم
Q3	"When I eat oranges, I enjoy putting the slices into my mouth and suck the orange juice out of the slices instead of just chewing the slices right away"	3- عند تناول البرتقال استمتع بوضع شرائح البرتقال في فمي و مصها أكثر من وضعها في فمي و مضغها مباشرة
Q4	"When I eat breakfast cereals, I usually let them soften quite a bit in the milk before I eat them, as opposed to eating them straight away"	4- عند اكل رقائق الذرة (الكورن فليكس) اتركها مغموسة في الحليب قليلا لتصبح طرية قبل اكلها بدلا من اكلها مباشرة بعد وضعها في الحليب
Q5	"When I eat chocolate, I usually prefer chocolate with a good chewing texture over chocolate that easily melts in the mouth"	5- من حيث القوام, عند تناول الشوكولاته افضل الشوكولاته التي لها قوام قابل للمضغ (براوني مثلا) اكثر من الشوكولاته التي تذوب بسهولة في الفم (شوكولا سادة مثلا)
Q6	"When I eat fruits, I usually prefer crunchy fruits like fresh apples over more chewy fruits that I can chew on like pineapple or strawberries"	6- من حيث القوام, عند تناول الفاكهة, عادة ا ما افضل الفاكهة الصلبة (المقرمشة) كالتفاح الطازج على الفاكهة القابلة للمضغ كالاناناس او الفراولة
Q7	"When it comes to chocolate, I usually prefer chocolate that is hard enough to suck on over chocolate that quickly melts in my mouth"	7- عندما يتعلق الموضوع بالشوكولاته من حيث القوام, افضل الشوكولاته الصلبة التي يمكن مصّها اكثر من تلك التي تذوب بسرعة في الفم
Q8	"I usually prefer soft and smooth fruits like ready to eat bananas and ripe peaches over hard and crunchy fruits like fresh apples and pears"	8- من حيث القوام, عادة ما افضل اكل الفواكه الطرية و اللينة كالموز و المشمش الناضج اكثر من الفاكهة الصلبة (المقرمشة) مثل التفاح الطازج او الاجاص
Q9	I usually prefer the texture of soft fresh white pita bread over the texture of crispbread and Crackers	9- افضل عادة قوام الخبز الأبيض الساخن الطري على الخبز المقرمش او الكعك/البسكويت المالح

**Q10**	**“When I eat ice cream, I eat it right out of the freezer instead of letting it thaw a little”**	10- عندما اتناول البوظة ,اتناولها مباشرة من الثلاجة بدلا من تركها تذوب قليلا قبل الاكل
**Q11**	**“I usually prefer to suck on hard candy until they are paper thin instead of crunching them after a short while”**	11- افضل عادة مص قطع الحلوى الصلبة (الملبس) حتى تصبح رقيقة كالورقة اكثر من مضغها مباشرة بعد وضعها بالفم
**Q12**	**“I enjoy to eat foods that are smooth and easily spreads in my mouth like puddings and ice cream”**	12- استمتع عند أكل الاطعمة الطرية و التي يسهل نشرها/فردها/توزيعها في الفم مثل المهلبية, الكستر او البوظة
**Q13**	**“When I eat cake, I usually prefer a chewy cake like brownie instead of a crunchy cake like Biscuits”**	13-من حيث القوام, عندما اكل الكيك افضل الكيك الذي له قوام قابل للمضغ مثل البراوني اكثر من البسكويت المقرمش
**Q14**	**“When I eat snacks, I usually prefer snacks that make a crunchy sound when I chew them like potato chips”**	14- عند تناول الاشياء الخفيفة (السناك) افضل تلك التي يصاحبها صوت قرمشة عند مضغها كرقائق البطاطا (الشيبس) المقرمشة
**Q15**	**“I usually prefer carbonated soft drinks like Coca-Cola over non-carbonated soft drinks like Lemonade”**	15- افضل عادة المشروبات الغازية مثل الكوكاكولا او البيبسي اكثر من المشروبات و العصائر غير الغازية كعصير الليمون
**Q16**	**“When I eat sweets, I usually prefer chocolate that easily melts in my mouth over hard candy that I would need to suck on”**	16- عند أكل الحلويات, من حيث القوام افضل عادة الشوكولاته التي تذوب بسهولة في الفم اكثر من الحلوى الصلبة (الملبس) الذي احتاج ان امصها
**Q17**	**“When I compare myself to my friends and family, I often find myself chewing my foods at a faster pace than them”**	17- عندما اقارن نفسي بأ صدقائي و عائلتي ألاحظ انني امضغ طعامي بشكل اسرع منهم
**Q18**	**When I eat roasted nuts decorating a dish of rice, I always prefer eating the roasted and crunchy nuts when they are fresh rather than eating them the second day when they become softened.**	18- عند اكل المكسرات التي يزين بها الطبق طبق الارز, افضل اكلها عندما تكون طازجة و مقرمشة اكثر من اكلها في اليوم التالي عندما تصبح طرية

**Q19**	**"I often find myself chewing foods on one side of the mouth only"**	19- غالبا ما اجد نفسي امضغ الطعام على جهة واحدة من الفم
**Q20**	**"I often experience difficulties in chewing when I eat tough foods like e.g., tough meat or wine gum"**	20- غالبا ما اواجه صعوبة بالمضغ (ألم أو تعب) عندما اكل الاطعمة القاسية مثل اللحم او حلوى الجيلاتين
**Q21 (Photo Based)**	**Please look at the picture and state to what extent you agree with the following statement:<br>I find great joy and pleasure in consuming products like these that have a good chew (Please consider all four products as a group, and consider your liking as an average for all four products). Illustrations included chewy strawberries, jelly gums, brownies, and chewy biscuits.**	21- الرجاء النظرالى الصور التالية و اعلامنا الى اي مدى توافق على الجملة الاتية: استمتع في تناول هذه المجموعة من الاطعمة التي يمكن مضغها (انظرالى هذه الاطعمة كمجموعة واحدة و قرر اعجابك بقوام الاطعمة الاربعة معا الاطعمة هي فراولة مطاطية او ذو قوام قابل للمضغ (تحتاج الى العلك), حلوى مطاطية (جيلاتينية), براونيز, بسكويت ذو قوام قابل للمضغ)
**Q22 (Photo Based)**	**Please look at the picture and state to what extent you agree with the following statement:<br>I find great joy and pleasure in consuming products like these that have a good crunch (Please consider all three products as a group, and consider your liking as an average for all three products). Illustrations included are raw carrots, crunch apples, and peanuts coated with chocolate illustrated with photos.**	22- الرجاء النظر الى الصور التالية و اعلامنا الى اي مدى توافق على الجملة الاتية: استمتع في تناول هذه المجموعة من الاطعمة المقرمشة (انظر الى هذه الاطعمة كمجموعة واحدة و قرر مدى اعجابك بقوام الاطعمة الثلاثة معا, الاطعمة هي جزر طازج , تفاح طازج, فول سوداني مغلف بالشوكولاتة)
**Q23 (Photo Based)**	**"Please look at the picture and state to what extent you agree with the following statement:<br>I find great joy and pleasure in consuming products like these that I can suck on until they dissolve (Please consider all four products as a group, and consider your liking as an average for all four products)". Illustrations include hard candy, mints, orange slices, hard chocolate.**	23- الرجاء النظر الى الصور التالية و اعلامنا الى اي مدى توافق على الجملة الاتية: استمتع في تناول هذه المجموعة من الاطعمة التي يمكن مصها طويلا حتى تذوب (انظر الى هذه الاطعمة كمجموعة واحدة و قرر مدى اعجابك بقوام الاطعمة الاربعة معا)
**Q24 (Photo Based)**	**Please look at the picture and state to what extent you agree with the following statement:<br>I find great joy and pleasure in consuming products like these that I can smoosh, and I even smoosh foods that I can chew (Please consider all three products as a group, and consider your liking as an average for all four products)". Illustrations include banana, chocolate pudding, ice cream.**	24- الرجاء النظر الى الصور التالية و اعلامنا الى اي مدى توافق على الجملة الاتية: استمتع في تناول هذه المجموعة من الاطعمة الطرية التي يمكن فردها و توزيعها بالفم (انظر الى هذه الاطعمة كمجموعة واحدة و قرر مدى اعجابك بقوام الاطعمة الثلاثة معا, الاطعمة هي موز, مهلبية شوكولاته, بوظة)

The blue highlighted row indicates the text-based question was slightly modified to include food items that were more familiar to our study sample.

Changes to Q9: I usually prefer the texture of soft whole-grain bread over the texture of crispbread and crackers (soft whole-grain bread was replaced with soft fresh white pita bread which is known to have a chewy texture in Jordan)

Changes to Q18: When I eat toppings on yoghurt products, I always prefer crunchy muesli and I avoid eating yoghurts where the muesli has softened was replaced with When I eat roasted nuts decorating a dish of rice, I always prefer eating the roasted and crunchy nuts when they are fresh rather than eating them the second day when they become softened.

The yellow highlighted row indicates the photo-based question was slightly modified to include a food item that was more familiar to our study sample or to remove a food item that was not familiar to our study sample. (Q21 replaced whole grain bread with brownies/ Q22 deleted crunchy granola/ Q24 deleted oatmeal)

“ ” Questions in quotation marks were quoted from Cattaneo et al *

** The POPB questionnaire used in this study was adapted from Food Quality and Preference, Volume 80, Camilla Cattaneo, Jing Liu, Anne C. Bech, Ella Pagliarini, Wender L.P. Bredie, Cross-cultural differences in lingual tactile acuity, taste sensitivity phenotypical markers, and preferred oral processing behaviors, 103803, 2020 with permission from Elsevier.
