# Supplementary material for: Oral processing behavior and dental caries; an insight into a new relationship
Source: PLoS One. 2024 Jul 2;19(7):e0306143. doi: 10.1371/journal.pone.0306143 (PMC11218957; doi:10.1371/journal.pone.0306143)
Supplement: Attachment — Submitted filename: Response to Reviewers.docx [file pone.0306143.s004.docx]

Melanie Alazzam

Corresponding Author

Assistant Professor

Department of Oral Medicine and Oral Surgery

Faculty of Dentistry

Jordan University of Science and Technology

P.O. Box: 3030, Irbid 22110, Jordan

Telephone: (+962)2-7201000 Ext: 23558

Email address: mfalazam@just.edu.jo

Dr. Hadi Ghasemi

Academic Editor

PLOS ONE

22.2.2024

Dear Dr. Ghasemi,

We would like to thank our reviewers for taking the time and effort to review this work. Their feedback is highly appreciated, and it has truly contributed to improving the quality of our manuscript. In what follows, we addressed each comment raised. In general, we made some modifications to the tone of the manuscript to ensure that our discussion and conclusions accurately reflect the findings of the study, while taking into consideration the limitations of our research.

Looking forward to hearing from you!

Sincerely,

Melanie Alazzam

| **Reviewer** | **Comment Number** | **Reviewer’s Comment** | **Authors’ Reply** | **Author’s Action**  **(Line numbers to be viewed while track changes mode is on)** |
| --- | --- | --- | --- | --- |
| Reviewer (1) | 1 | Please include the POPB questionnaire | We appreciate your feedback. We have included the POPB questionnaire per your request. We sought permission from Elsevier to reuse the questionnaire and the translated version in our future publication. The license number is 5662390874243. We did not include the questionnaire previously due to uncertainty about the terms of the permission granted to us. Further correspondence with Elsevier clearly stated that we can reuse and include the POPB questionnaire, and the Arabic version as shown in the uploaded supplementary file. | **A supplementary data file was uploaded “S1 file”** |
| Reviewer (1) | 2 | Using a translated questionnaire for study requires standardization of the translated version (translation, back translation, expert opinion...). Please explain more if you have standardized the question. | Thank you for highlighting this point. The questionnaire was forward translated into the Arabic language by a committee of three competent bilingual speakers. The committee included a linguist (Dr. Aziz Jaber) and two clinicians, Prof. Suhad Aljundi and Dr. Mohammad Atieh. A training session was conducted in which research assistants were introduced to the aims of the study and the POPB questionnaire. Next, the questionnaire was distributed to a pilot sample of 23 participants. Each participant filled out the questionnaire, and comments or questions were recorded by the accompanying research assistant. Another meeting with the research assistants was held to address the recorded comments/feedback regarding the Arabic version of the questionnaire. Based on responses from the pilot sample and the revision done by two of the committee members, two text questions and three photo-based questions were slightly modified. The modifications aimed to include/remove food items that were more familiar/unfamiliar to our study group (details are included in the supplementary data file)  Although back translation is commonly used in the literature as a tool to ensure the accuracy of the translated questionnaire, its use and efficacy as a standard assessment method are controversial. Some researchers have noted inconsistency in the detection of presumed flaws when more individuals are employed to compare back translation with the original text. While back translation can uncover problems, it also leads to a significant number of false alarms. Moreover, a significant number of issues persist, as demonstrated by Behr [1] and Colina [2]. Other researchers argue that using back translation as a quality assessment tool in multilingual survey research is now deemed obsolete or on its way to becoming so, although it is still widely practiced. It is argued that back translation may still be useful as a documentation tool, not as a quality control tool[3].  **Regarding the questionnaire used in the study, most of its items consist of repetitive sentences with food item variables. The food items in English have unique equivalent terms in Arabic, rendering back translation less effective. Instead, we chose to standardize the translated questionnaire using a more accuracy-ensuring method**. | Materials and methods/ Line 195:  **“and the experts’ opinion” as this was done but was not clearly stated in the first draft of the manuscript.**  (highlighted in green) |
| Reviewer (2) | 1 | Initial lesions were not recorded, and radiographic imaging was not used, why? As the authors must know, without radiographs there will be problems with false negative outcomes. I know this is discussed in the discussion, but this is truly a major issue. | Thank you for highlighting this point. We acknowledge the relevance of this matter. We appreciate that this might be a major issue if it was done for one of the groups and if such a compromise was associated with an overestimation of dental caries. Below we provide the reasons why initial enamel lesions were not recorded and why radiographs were not taken.  Initial lesions were not recorded as we chose the DMFS index to assess caries experience. The index was chosen since it's time-saving and easy to apply **[4, 5].** When assessing caries experience using the DMFS, the WHO guidelines were followed which exclude early non-cavitated enamel lesions. As we designed the study, we took into consideration that using the DMFS index would underestimate caries experience compared to other methods such as the ICDAS and CAST**[4]**. This would further challenge our hypothesis and would make it harder to detect a difference in caries experience among the groups.  Radiographs were not taken for financial and participant-related reasons. This research was a non-funded project. Furthermore, we collected many variables including dental caries experience and the plaque index. Recording the plaque index is time-consuming and tiring for the participant. Taking radiographs was compromised to make the data collection session shorter and to reach a larger sample size. Such a compromise was anticipated to underestimate caries experience and would further challenge our hypothesis. The underestimation would make it harder to detect a difference in caries experience among the groups. However, a significant difference in caries experience was detected between the different oral processing behaviors.  Nevertheless, we believe that using the DMFS index with no bitewing radiographs was useful for the purpose of exploring a potential relationship similar to other studies investigating new associations with dental caries**[6-9].** | Discussion/Line 392: **Initial lesions and radiographs were discussed to explain the observation pertinent to the “D” component of the DMFS.**  (highlighted in yellow)  Discussion/Line 460 : **Initial lesions and radiographs were added as new limitations to our study.**  (highlighted in yellow) |
| Reviewer (2) | 2 | DMFT was significantly higher in one of the groups, however, when looking at the individual aspects, only the M = missing was significantly higher. Is it a correct conclusion to draw that dental caries has a correlation with eating behaviors in this study? For me, it is rather oral health and not specifically dental caries that can be discussed since decayed and filled were not significantly higher in one of the groups. The limitation of the DMFT-index is that the M = missing part is always unknown regarding the etiology. | We value your concern. Indeed, we had similar concerns about our findings which were included in our discussion. Although we provided an evidence-based explanation for our findings, your feedback (in comments 2,3,4,5) enabled us to gain a deeper understanding of our data and implement modifications that accurately represent the insights we’ve discovered. Therefore, we made several changes in the manuscript that are detailed in the author’s actions column. In general, these amendments included the title, abstract, discussion and conclusions. | Title page: **added “an insight into” the title.**  (highlighted in yellow)  Abstract/ Line 39: **changed the word “determine” to “explore if a relationship exists” in the aims of the study.**  (highlighted in yellow)  Abstract/Line 47: **deleted the sentence “The preferred oral processing behavior did not show any predilection for sex(P=0.409) or age(P=0.053).”**  (highlighted in yellow)  Abstract/Line 50: **deleted** **the sentence “ In contrast, the mean total plaque score was similar 0.61(± 0.60) for both groups (P=0.964). Plaque accumulation levels did not vary significantly per quadrant or arch**”.  (highlighted in yellow)  Abstract/ Line 52: **added the sentence “exhibited a significantly higher mean score for the “M” component (P <0.001) while no significant difference in the mean score of the “D”(P= 0.076), and “F”(P=0.272) components was observed when compared to FPL**.”  (highlighted in yellow)  **Changes in lines 47,50,52 were made to display outcomes that are more relevant to our conclusions.**  (highlighted in yellow)  Abstract/Line 55**: modified the conclusion for a better representation of the insights discovered.**  (highlighted in yellow)  Introduction/ Line 143  Introduction/ Line 145  **Amendments were made in the aims section that explain the aims and their boundaries in a more accurate manner**.  (highlighted in yellow)  Discussion / Line 371  Discussion / Line 375  Discussion /Line 383  **Confirmation of the actual aims of the study and exclusion of other aims that could be mistakenly perceived by the reader.**  (highlighted in yellow)  Discussion /Line 392-440  **This part was re-written partly to make sure the paper is not favoring certain direction for the discovered relationship but rather is encouraging the reader to consider all aspects of such potential association.**  (highlighted in yellow)  Discussion / Line 465  **The limitations of the DMFS , ,specifically the M component, was addressed. We discussed the biases inherent within the “M” component to keep the reader aware of these limitations when examining our data.**  (highlighted in yellow)  Discussion / Line 516  **Modified the conclusion for a better representation of the insights discovered.**  (highlighted in yellow) |
| Reviewer (2) | 3 | In the manuscript, the aspect of seeking dental care is discussed, and how this affects the results. The question of seeking dental care, and specifically asking about why and what kind of care (only emergency, regular check-ups, filling therapy) should have been included in the questionnaire. | We appreciate highlighting this matter. Indeed, evidence suggests that the number of missing teeth may vary depending on the reason for seeking dental care. For instance, there was an association between seeking dental care when having symptoms versus not having symptoms and the number of missing teeth **[10, 11]**. Therefore, we added this as a limitation to our study while keeping in mind the complex nature of dental caries and the difficulty of addressing all confounders in a single study**[12]**. | Discussion / Line 482  **This was added as a new limitation to our research.**  (highlighted in yellow) |
| Reviewer (2) | 4 | The largest limitation in this manuscript is that the fluoride aspect has not been accounted for. Toothbrushing was included in the questionnaire, but not fluoride concentrations in toothpaste, toothpaste behaviors (such as amounts of fluoride toothpaste used, rinsing with water during or after brushing), or usage of additional fluoride products. The usage of fluoride in the treatment and prevention of dental caries is of great importance and probably affects the results in this study. This is not even discussed in the manuscript, why? | We acknowledge your concern. Despite collecting information about the participant’s oral hygiene practices, certain details related to the use of fluoride in oral hygiene practices were not acquired. We acknowledge the anticaries role of fluoride and the effect of different tooth brushing behaviors on dental caries experience. Therefore, this point was addressed by including it as a new limitation to our research. | Discussion / Line 478  **This was added as a new limitation to our research**.  (highlighted in yellow) |
| Reviewer (2) | 5 | Additionally, saliva secretion rates, and buffer capacity has not been included either which is a limitation since this affects the caries risk. | Thank you for pointing out this factor. Indeed, several studies demonstrate a relationship between dental caries and certain salivary parameters. However, dental caries is a multifactorial disease, adjusting for all confounders in one study is not feasible**[12]**. Compared to other published work**[13, 14],** our research adjusted for a large group of confounders (age, sex, BMI, medical problems, medications, dental visits, brushing and flossing frequency, consumption frequency of sugary drinks, candies, snacks, and sugary baked item, smoking status, participant’s education level, mothers’ education level, income level and the plaque index). However, we were keen to keep the reader informed of the limitations raised by reviewer (2) as to be addressed in future studies confirming/contradicting our observations. | Discussion / Line 487  Added “and buffer capacity”  (highlighted in yellow) |
| Reviewer (2) | 6 | Regarding supplementary information, I cannot see the pictures used in the questionnaire about food preferences, and I cannot find the questionnaire used for collecting the general information. | We appreciate your feedback. We have included the POPB questionnaire per your request. We sought permission from Elsevier to reuse the questionnaire and the translated version in our future publication. The license number is 5662390874243. We did not include the questionnaire previously due to uncertainty about the terms of the permission granted to us. Further correspondence with Elsevier clearly stated that we can reuse and include the POPB questionnaire, and the Arabic version as shown in the uploaded supplementary file.  There were 4 photo-based questions (Q21-Q24). We did not include the pictures as the permission was granted to us to reuse the questionnaire in our manuscript in the format presented in the supplementary file. However, food items presented in these pictures are clearly stated in each of the photo-based questions such as (raw carrots, apples, and peanuts coated with chocolate etc). Kindly note that the pictures are available in the original questionnaire as part of the supplementary data and can be found using the following link: [Cross-cultural differences in lingual tactile acuity, taste sensitivity phenotypical markers, and preferred oral processing behaviors - ScienceDirect](https://www.sciencedirect.com/science/article/pii/S0950329319304653?casa_token=VmjjOUwbHmkAAAAA:e9Me7nqwOr9dVmf-XgfzaLj9ouBCCt8ToA3doKrl20OmL2RfBvUWSkHMBZSZUo1sFoBHlmQRqA) | **A supplementary data file was uploaded “S1 file”.** |
| Reviewer (3) | 1 | Would it have been meaningful to include patients with minimal missing teeth or possible none to eliminate this confounding factor? | Thank you for highlighting this point. We agree with you. Studying this in a sample of dentate individuals will improve our understanding of this relationship. It will also help us overcome the potential unwanted overestimation of the “M” component due to the different biases discussed in the manuscript. Accordingly, this point was added to the limitations of the study. | Discussion / Line 470 & 526  **This was added as a new limitation to our research and to conclusions.**  (highlighted in light blue) |
| Reviewer (3) | 2 | With the information you have collected would it not have been more meaningful to perform caries risk assessment? I encourage you to present it in caries risk format if you are able to. It would simplify data presentation and make comparisons with literature easier since most studies are reporting in that way. | Thank you for the suggestion. We agree with your point. However, doing this retrospectively seemed to be unfeasible since certain factors in the available caries risk assessment forms/tools were not included in our questionnaire. The ADA caries risk assessment form, Cariogram, AAPD CRA Tool, and CAries Management By Risk Assessment (CAMBRA) are an example. Nevertheless, we are planning to use the caries assessment form/tools in the near future to highlight and understand the relationship between oral processing and dental caries. | -------- |
| Reviewer (3) | 3 | What is the importance of collecting plaque indices since its relationship with dental caries has proven to be weak | We appreciate asking about this point. When conducting this study, we had the hypothesis that differences in the preferred oral processing behavior are potentially associated with differences in dental plaque levels. Based on the available evidence**[15-18]**, we assumed that differences in dental plaque levels may contribute to differences in caries experience. Accordingly, we anticipated that FPL, who preferred using their teeth during oral processing and preferred hard food textures would demonstrate lower plaque levels compared to SPL. This hypothesis was derived from a study demonstrating changes in dental plaque levels when chewing an apple**[19, 20]**. A food texture that is most likely not preferred by SPL group **[21]**.  Your feedback (comments 1 and 3) were helpful in changing the tone of our study and | Discussion / Line: 447  **The purpose of collecting dental plaque and its related results were**  **discussed.**  (highlighted in light blue) |

**References:**

1.	Behr D. Assessing the use of back translation: the shortcomings of back translation as a quality testing method. International Journal of Social Research Methodology. 2017;20(6):573-84. doi: 10.1080/13645579.2016.1252188.

2.	Colina S, Marrone N, Ingram M, Sánchez D. Translation Quality Assessment in Health Research: A Functionalist Alternative to Back-Translation. Evaluation & the Health Professions. 2017;40(3):267-93. doi: 10.1177/0163278716648191. PubMed PMID: 27207929.

3.	Son J. Back translation as a documentation tool. Translation & Interpreting: The International Journal of Translation and Interpreting Research. 2018;10(2):89–100.

4.	Castro ALS, Vianna MIP, Mendes CMC. Comparison of caries lesion detection methods in epidemiological surveys: CAST, ICDAS and DMF. BMC Oral Health. 2018;18(1):122. doi: 10.1186/s12903-018-0583-6.

5.	Castro ALS, Vianna MIP, Mendes CMC. The knowledge and use of population-based methods for caries detection. BMC Oral Health. 2018;18(1):153. doi: 10.1186/s12903-018-0612-5.

6.	Shen A, Bernabé E, Sabbah W. The bidirectional relationship between weight, height and dental caries among preschool children in China. PLOS ONE. 2019;14(4):e0216227. doi: 10.1371/journal.pone.0216227.

7.	Vergnes J-N, Kaminski M, Lelong N, Musset A-M, Sixou M, Nabet C, for the Eg. Frequency and Risk Indicators of Tooth Decay among Pregnant Women in France: A Cross-Sectional Analysis. PLOS ONE. 2012;7(5):e33296. doi: 10.1371/journal.pone.0033296.

8.	Iwasaki M, Kakuta S, Ansai T. Associations among internet addiction, lifestyle behaviors, and dental caries among high school students in Southwest Japan. Sci Rep. 2022;12(1):17342. doi: 10.1038/s41598-022-22364-0.

9.	Olczak-Kowalczyk D, Krämer N, Gozdowski D, Turska-Szybka A. Developmental enamel defects and their relationship with caries in adolescents aged 18 years. Sci Rep. 2023;13(1):4932. doi: 10.1038/s41598-023-31717-2.

10.	Hawley GM, Holloway PJ, Davies RM. Dental health status associated with documented dental attendance patterns in adolescents. Community dental health. 1997;14(1):22-4. PubMed PMID: 9114545.

11.	Lopez R, Baelum V. Factors associated with dental attendance among adolescents in Santiago, Chile. BMC Oral Health. 2007;7(1):4. doi: 10.1186/1472-6831-7-4.

12.	Nascimento GG, Chaffee BW. Causal Inference in Oral Health Epidemiology. In: Peres MA, Antunes JLF, Watt RG, editors. Oral Epidemiology: A Textbook on Oral Health Conditions, Research Topics and Methods. Cham: Springer International Publishing; 2021. p. 267-78.

13.	Peng SM, Wong HM, King NM, McGrath C. Association between Dental Caries and Adiposity Status (General, Central, and Peripheral Adiposity) in 12-Year-Old Children. Caries research. 2013;48(1):32-8. doi: 10.1159/000351690.

14.	Campus G, Cagetti MG, Senna A, Blasi G, Mascolo A, Demarchi P, Strohmenger L. Does Smoking Increase Risk for Caries? A Cross-Sectional Study in an Italian Military Academy. Caries research. 2011;45(1):40-6. doi: 10.1159/000322852.

15.	Laajala A, Pesonen P, Anttonen V, Laitala M-L. Association of Enamel Caries Lesions with Oral Hygiene and DMFT among Adults. Caries research. 2019;53(4):475-81. doi: 10.1159/000497358.

16.	Leroy R, Bogaerts K, Martens L, Declerck D. Risk factors for caries incidence in a cohort of Flemish preschool children. Clin Oral Investig. 2012;16(3):805-12. doi: 10.1007/s00784-011-0579-y.

17.	Warren JJ, Weber-Gasparoni K, Marshall TA, Drake DR, Dehkordi-Vakil F, Kolker JL, Dawson DV. Factors Associated with Dental Caries Experience in 1-Year-Old Children. J Public Health Dent. 2008;68(2):70-5. doi: <https://doi.org/10.1111/j.1752-7325.2007.00068.x>.

18.	Declerck D, Leroy R, Martens L, Lesaffre E, Garcia-Zattera M-J, Broucke SV, et al. Factors associated with prevalence and severity of caries experience in preschool children. Community dentistry and oral epidemiology. 2008;36(2):168-78. doi: <https://doi.org/10.1111/j.1600-0528.2007.00385.x>.

19.	Schneider HG, Knieknecht I. [Reduction of dental plaque by apples and chewing gum]. Nahrung. 1986;30(9):907-12. PubMed PMID: 3796719.

20.	Rubido S, García-Caballero L, Abeleira MT, Limeres J, García M, Diz P. Effect of chewing an apple on dental plaque removal and on salivary bacterial viability. PLOS ONE. 2018;13(7):e0199812. doi: 10.1371/journal.pone.0199812.

21.	Jeltema M, Beckley J, Vahalik J. Food texture assessment and preference based on Mouth Behavior. Food Quality and Preference. 2016;52:160-71. doi: <https://doi.org/10.1016/j.foodqual.2016.04.010>.
