# Supplementary material for: Oral processing behavior and dental caries; an insight into a new relationship
Source: PLoS One. 2024 Jul 2;19(7):e0306143. doi: 10.1371/journal.pone.0306143 (PMC11218957; doi:10.1371/journal.pone.0306143)
Supplement: Attachment — Submitted filename: Response to Reviewers.docx [file pone.0306143.s005.docx]

Melanie Alazzam

Corresponding Author

Assistant Professor

Department of Oral Medicine and Oral Surgery

Faculty of Dentistry

Jordan University of Science and Technology

P.O. Box: 3030, Irbid 22110, Jordan

Telephone: (+962)2-7201000 Ext: 23558

Email address: mfalazam@just.edu.jo

Dr. Hadi Ghasemi

Academic Editor

PLOS ONE

4.16.2024

Dear Dr. Ghasemi,

We would like to thank you and the reviewers for your interest in our work. We value your feedback and your role in ensuring the quality and integrity of any original research to be published at PLOS One. We would like to thank reviewer 4 for the recommendations which were helpful in further improving our manuscript. In what follows, we addressed each comment raised. In general, we made a few amendments to the methods, results, discussion, and references sections. Moreover, we corrected a few typos, grammatical, punctuation marks, and spelling errors that were discovered while reviewing the manuscript.

Looking forward to hearing from you!

Sincerely,

Melanie Alazzam

| **Editor/ Reviewer** | **Comment Number** | **Reviewer’s Comment** | **Authors’ Reply** | **Author’s Action**  **(Line numbers to be viewed while track changes mode is on)** |
| --- | --- | --- | --- | --- |
| Dr. Ghasemi | 1 | In my perspective, your research warrants attention and publication due to the originality of its concept. In the last paragraph of the M&M (lines 255-256), however, I noticed that you mentioned about a GLM analysis but I did not find any related table or text in the result section in this regard. As you have a lot of confounding variables in your study, it is essential to perform a multivariable analysis like a binary logistic regression. Please, add a table and explain it in the text of the result section. | We value your request.  The following was the response from our statistician Dr. Yousef Khader “The GLM procedure offers regression analysis for a single continuous dependent variable, influenced by one or more factors and/or variables. This analytical approach incorporates multiple predictor variables simultaneously to elucidate their relationships with the response variable. In Table 3, the GLM results, constituting regression analysis, reveal differences in the average DMFS index and PI between the two oral processing groups. These differences are observed after adjustment for various covariates, including age, sex, BMI, medical problems, medications, dental visits, brushing and flossing frequency, consumption frequency of sugary drinks, candies, snacks, and sugary baked item, smoking status, participant’s education level, parents’ education level, and income level.  We have revised the text to reflect that the GLM represents a form of regression analysis and to emphasize that the findings presented in Table 3 are adjusted for potential confounders and additional predictors.  The binary (binomial) logistic regression analysis cannot be done since the variables are quantitative (not dichotomous).[1, 2]”  Kindly note we are willing to fulfill any recommendations/requests if our response does not address your concern.  1. Ranganathan P, Pramesh CS, Aggarwal R. Common pitfalls in statistical analysis: Logistic regression. Perspect Clin Res. 2017;8(3):148-51. doi: 10.4103/picr.PICR_87_17. PubMed PMID: 28828311; PubMed Central PMCID: PMCPMC5543767.  2. Faizi N, Alvi Y. Chapter 11 - Regression and multivariable analysis∗∗For datasets, please refer to companion site: <https://www.elsevier.com/books-and-journals/book-companion/9780443185502>. In: Faizi N, Alvi Y, editors. Biostatistics Manual for Health Research: Academic Press; 2023. p. 213-47. | **Amendments are highlighted in yellow.**  **Line 259**  **Line 321**  **Line 334**  **Line 340 (Title of Table 3)**  **Line 353** |
| Reviewer (4) | 1 | • Both the quality and data presentation of this manuscript are acceptable and of great importance to clinicians as well as patients.  • The manuscript expands our knowledge about oral processing behavior and oral diseases including dental caries | Thank you for taking the time to read our paper. We value your appreciation of the significance and the potential impact of this research. |  |
| Reviewer (4) | 2 | The title should be revised and reduced its characters ( precise & and informative) | We value your recommendation. During the process of choosing a title for our manuscript, we strictly followed PLOS One guidelines pertinent to the full title. The guidelines requested a title that is no more than 250 characters in length. Therefore, our title included 67 characters with no spaces (79 characters with spaces). Moreover, we intended to include the variables of interest in our title (**oral processing behavior, and dental caries**) and the most relevant finding of this research (the existence of **a new relationship** between these variables). Since caries is a multifactorial disease, we avoided confirming this relationship and chose words that would make the title more representative of the current level of evidence provided by this manuscript. Therefore, we added the words “**an insight into**”. These lead us to the title “**Oral processing behavior and dental caries; an insight into a new relationship**” |  |
| Reviewer (4) | 3 | The abstract should reflect the content of the article and must be within the range of 250-300 words. | Yes, we agree. PLOS One requires an abstract that does not exceed 300 words. Though our abstract looks long, using the word count function in Microsoft Word the abstract is 299 words in length.  We were keen to include the rationale and the aim of the study. We tried our best to choose the study’s relevant findings. Based on previous feedback, we made changes to the results section to make it more representative of our work. We presented the main findings of the study and the breakdown of the different components of the DMFS index. These were chosen as they were discussed comprehensively in our manuscript. We avoided shaping the reader's thoughts of this relationship by confirming that “ The current findings provide new insight into a possible relationship between the preferred oral processing behavior and an individual’s caries experience. A relationship in which the preferred oral processing behavior can potentially affect and/or be affected by the dental caries experience. “ |  |
| Reviewer (4) | 4 | Three to ten keywords representing the main content of the article | We appreciate shedding light on this matter. Indeed, we thought that the keywords would be part of the manuscript during the submission process. However, the sample title page at PLOS One indicated that the keywords are not included as part of the manuscript. Moreover, PLOS One guidelines indicated that the keywords are included via the submission system. <https://journals.plos.org/plosone/s/file?id=3fac/PLOS%20Affiliations%20Formatting%20Guidelines.pdf>  We have chosen the following keywords previously: “oral processing; oral processing behavior, questionnaire; Caries; Dental caries; firm processing likers; soft processing likers, cross-sectional; mouth behavior, relationship”  However, we made some changes to the keywords so that they would be more precise and represent the main content of the article. | **Deleted the keywords “cross-sectional and questionnaire”.**  The amended keywords are: **“oral processing, mouth behavior, soft processing likers, firm processing likers, caries, relationship ”**  The amended keywords are available at PLOS One Submission system and are not part of the manuscript according to the Journal’s guidelines. |
|  | 5 | Although the results are impressive and interesting but need to be revised to be more informative by precise presentation of your findings. | Thank you for your interest in the findings of the study. We agree with you. We noticed that the table (Table 3) and the data representing the GLM analysis were not sufficiently informative and precisely represented. Therefore, we made some changes that will enable the reader to understand the findings of the GLM analysis and its interpretation. | **Amendments are highlighted in yellow.**  **Line 259**  **Line 321**  **Line 334**  **Line 340 (Title of Table 3)**  **Line 353** |
|  | 6 | The statements in discussion are acceptable but more paragraphs about the justification of your findings and comparison with other recent relevant studies. | We value your suggestion. It helped us identify other important findings related to our study. Our study demonstrated that the preferred oral processing behavior was independent of the individual’s age and sex. This finding was compared to other studies.  Moreover, another paragraph was added which discusses other means by which the preferred oral processing behavior may increase an individual’s caries experience based on the available evidence. | The new paragraphs were highlighted in yellow.  **Line 389**  **Line 445** |
|  | 7 | Up-to-date references should be kept in your reference list and the old ones should be omitted. | Thank you for pointing this out. We tried our best to update the reference list. We noticed that all references directly related to oral processing behavior are not older than 8 years. For all other cited references, we tried our best to update the references so that they will not be older than 10 years. However, a few older references were kept for different reasons. Below is a list of all references older than 10 years and the action taken regarding each reference. An explanation is provided regarding each action.  We have included the older reference list to be able to follow the changes implemented in the latest version of the manuscript. The numbers mentioned below represent the numbers used in the older reference list (see below this table).    Regarding reference #1  The reference was replaced with a more recent reference covering the idea of the statement (Prakash; 2017).  Regarding references # 3, 5  The reference was kept. The notion of the presented statement was clearly stated in these references and was not found as clear in more recent references. The references are important for explaining the concept/idea of the statement to non-food/food technology scientists.  Regarding reference #6  The reference was replaced with a more recent reference covering the idea of the statement (Prakash; 2017).  Regarding reference #10  The reference was kept. The notion of the presented statement was clearly stated in these references and was not found as clear in more recent references. The references are important for explaining the concept/idea of the statement to non-food/food technology scientists  Regarding references #17, 18, 19  The references were kept. The references were crucial supporting evidence and /or explaining the available evidence. We have added a new reference (Mathur et al; 2018) that supports the statement. The reference was added to confirm that the evidence provided is still valid in the dental caries field.  Regarding reference # 22  This was the original research from which the global burden of dental caries was estimated and where dental caries was found to be the most prevalent health condition. Therefore, we kept the reference and added a recent reference (de Lima; 2024) that demonstrated that these numbers are still valid and used in recently published research.  Regarding reference # 26  The reference was replaced with two more recent references (Carmen A-D; 2021, Paiva; 2021) supporting the idea of the statement.  Regarding reference # 34  The reference was crucial supporting evidence and /or explaining the available evidence. Therefore, the reference was kept. We have added a new reference (Mathur et al; 2018) that supports the statement. The reference was added to confirm that the evidence provided is still valid in the dental caries field.  Regarding reference # 36  The reference was replaced with a more recent publication(Mathur et al; 2018) supporting the idea of the statement.  Regarding reference # 38  Important evidence supporting the relationship between eating time and dental caries experience. Recent publications were not present in indexed journals. Therefore, the reference was kept.  Regarding reference # 39  The reference was replaced with a more recent publication (Glabska et al; 2023) supporting the idea of the statement.  Regarding reference # 41  The reference was replaced with a more recent publication (Watanabe et al; 2023) supporting the idea of the statement.  Regarding reference # 42  The reference was replaced with a more recent publication (Peres et al; 2021) supporting the idea of the statement.  Regarding reference # 43  To our knowledge, this was the latest reference for the WHO guidelines pertinent to the DMFS index. The DMFS index was developed by the WHO.  Regarding reference # 44, 46  To our knowledge, this was the first paper to introduce the plaque index. Therefore, the papers were kept. In some other instances, these references were cited in addition to another more recent publication (Shafaie et al,2024) which indicates the index is still a valid and reliable tool to measure dental plaque levels.  Regarding reference # 47  The reference was replaced with a newer publication (Cengiz et al:2024).  Regarding reference # 51  The reference was kept for being a very useful review for the reader to refer to regarding the relation between dental status and mastication (oral processing).  Regarding reference # 54  The reference was replaced with a newer publication (Almotairy; 2021).  Regarding references # 55, 57, 58, 59  The references provided supportive evidence for the presented argument.  Regarding references # 62, 63  The notion of the presented statements was clearly stated in these references and is not found as clear in more recent references. This is a controversial topic.  Regarding reference# 70  The reference was removed. There were another 3 cited references that support the notion of the statement.  Regarding references # 74, 75  References were kept. They are part of a group of references that were provided to demonstrate that it is almost impossible to adjust for all confounders in a single study. We provided 2 examples from recent literature (Shen et al; 2019) and another 3 from older literature (Vergnes et al; 2012, Campus et al;2011, Peng et al; 2013) to show that this issue was and is still encountered in dental caries research.  Regarding reference # 77  The reference was replaced with newer publications (Creeth et al; 2016, Ishizuka et al; 2020).  Regarding references # 78, 79  The notion of the presented statement was clearly stated in these references and is not found as clear in more recent references. Therefore, they were kept.    Regarding references # 80, 81  The references were replaced with two more recent publications (Gachova; 2023, Negm; 2024) supporting the idea of the statement.  Regarding reference # 84  The reference was replaced with newer publications (Munoz et al; 2024, Alqahtani et al 2024)  Regarding reference # 87  The reference was replaced with newer publications (Sangalli et al; 2023, Laajala et al 2023)  Regarding reference # 89  The reference was replaced with newer publications (Zhang et al; 2020, Kakkar et al 2022)  Regarding reference # 94  This is the original work for the percentage provided. However, we added another recent reference showing that this information is still accepted (Steed et al; 2014).  Regarding reference # 95  The reference was replaced with newer publication (Vandeplas et al; 2020). | **Updated references were highlighted in yellow in the manuscript.** |

**Old List of References (For Comparison Purposes with the New Reference List)**

1.	Stieger M, van de Velde F. Microstructure, texture and oral processing: New ways to reduce sugar and salt in foods. Current Opinion in Colloid & Interface Science. 2013;18(4):334-48. doi: <https://doi.org/10.1016/j.cocis.2013.04.007>.

2.	Wang X, Chen J. Food oral processing: Recent developments and challenges. Current Opinion in Colloid & Interface Science. 2017;28:22-30. doi: <https://doi.org/10.1016/j.cocis.2017.01.001>.

3.	Chen J. Food oral processing—A review. Food Hydrocolloids. 2009;23(1):1-25. doi: <https://doi.org/10.1016/j.foodhyd.2007.11.013>.

4.	Depeyre A, Pereira B, Pham-Dang N, Barthélémy I, Hennequin M. Impairments in Food Oral Processing in Patients Treated for Tongue Cancer. Dysphagia. 2020;35(3):494-502. doi: 10.1007/s00455-019-10054-5.

5.	Foster KD, Grigor JMV, Cheong JN, Yoo MJY, Bronlund JE, Morgenstern MP. The Role of Oral Processing in Dynamic Sensory Perception. Journal of Food Science. 2011;76(2):R49-R61. doi: 10.1111/j.1750-3841.2010.02029.x.

6.	Prakash S, Tan DDY, Chen J. Applications of tribology in studying food oral processing and texture perception. Food Research International. 2013;54(2):1627-35. doi: <https://doi.org/10.1016/j.foodres.2013.10.010>.

7.	Laguna L, Barrowclough RA, Chen J, Sarkar A. New Approach to Food Difficulty Perception: Food Structure, Food Oral Processing and Individual's Physical Strength. Journal of Texture Studies. 2016;47(5):413-22. doi: <https://doi.org/10.1111/jtxs.12190>.

8.	Jeltema M, Beckley JH, Vahalik J. Importance of understanding mouth behavior when optimizing product texture now and in the future. Food Texture Design and Optimization2014. p. 423-42.

9.	Jeltema M, Beckley J, Vahalik J. Food texture assessment and preference based on Mouth Behavior. Food Quality and Preference. 2016;52:160-71. doi: <https://doi.org/10.1016/j.foodqual.2016.04.010>.

10.	J. Chen LE. Food oral processing: Fundamentals of eating and sensory perception. Food Oral Processing. Oxford: John Wiley & Sons; 2012. p. 157-76.

11.	Doyennette M, Aguayo-Mendoza MG, Williamson A-M, Martins SIFS, Stieger M. Capturing the impact of oral processing behaviour on consumption time and dynamic sensory perception of ice creams differing in hardness. Food Quality and Preference. 2019;78:103721. doi: <https://doi.org/10.1016/j.foodqual.2019.103721>.

12.	Jeltema M, Beckley J, Vahalik J. Model for understanding consumer textural food choice. Food Science & Nutrition. 2015;3(3):202-12. doi: 10.1002/fsn3.205.

13.	Cattaneo C, Liu J, Bech AC, Pagliarini E, Bredie WLP. Cross-cultural differences in lingual tactile acuity, taste sensitivity phenotypical markers, and preferred oral processing behaviors. Food Quality and Preference. 2020;80:103803. doi: <https://doi.org/10.1016/j.foodqual.2019.103803>.

14.	Liu J, Bech AC, Stolzenbach Waehrens S, Bredie WLP. Perception and liking of yogurts with different degrees of granularity in relation to ethnicity, preferred oral processing and lingual tactile acuity. Food Quality and Preference. 2021;90:104158. doi: <https://doi.org/10.1016/j.foodqual.2020.104158>.

15.	Chaffee O, Laura Montero M, Keast R, Ross CF. Oral acuity, particle size sensitivity, and food texture preferences in an older adult population. Food Quality and Preference. 2023;112:105031. doi: <https://doi.org/10.1016/j.foodqual.2023.105031>.

16.	Mathiesen SL, Mielby LA, Byrne DV, Wang QJ. Music to eat by: A systematic investigation of the relative importance of tempo and articulation on eating time. Appetite. 2020;155:104801. doi: <https://doi.org/10.1016/j.appet.2020.104801>.

17.	Kashket S, Zhang J, Van Houte J. Accumulation of Fermentable Sugars and Metabolic Acids in Food Particles that Become Entrapped on the Dentition. Journal of Dental Research. 1996;75(11):1885-91. doi: 10.1177/00220345960750111101. PubMed PMID: 9003236.

18.	Touger-Decker R, van Loveren C. Sugars and dental caries23. The American journal of clinical nutrition. 2003;78(4):881S-92S. doi: <https://doi.org/10.1093/ajcn/78.4.881S>.

19.	Bowen WH, Amsbaugh SM, Monell-Torrens SM, Brunelle J. Effects of Varying Intervals between Meals on Dental Caries in Rats. Caries research. 2009;17(5):466-71. doi: 10.1159/000260703.

20.	Pitts NB, Twetman S, Fisher J, Marsh PD. Understanding dental caries as a non-communicable disease. British Dental Journal. 2021;231(12):749-53. doi: 10.1038/s41415-021-3775-4.

21.	Giacaman RA, Fernández CE, Muñoz-Sandoval C, León S, García-Manríquez N, Echeverría C, et al. Understanding dental caries as a non-communicable and behavioral disease: Management implications. Front Oral Health. 2022;3:764479. Epub 20220824. doi: 10.3389/froh.2022.764479. PubMed PMID: 36092137; PubMed Central PMCID: PMCPMC9448953.

22.	Marcenes W, Kassebaum NJ, Bernabé E, Flaxman A, Naghavi M, Lopez A, Murray CJ. Global burden of oral conditions in 1990-2010: a systematic analysis. J Dent Res. 2013;92(7):592-7. Epub 2013/05/31. doi: 10.1177/0022034513490168. PubMed PMID: 23720570; PubMed Central PMCID: PMCPMC4484374.

23.	Kassebaum NJ, Bernabé E, Dahiya M, Bhandari B, Murray CJ, Marcenes W. Global burden of untreated caries: a systematic review and metaregression. J Dent Res. 2015;94(5):650-8. Epub 2015/03/06. doi: 10.1177/0022034515573272. PubMed PMID: 25740856.

24.	Cheng R, Shao J, Gao X, Tao C, Ge J, Liu X. Noninvasive Assessment of Early Dental Lesion Using a Dual-Contrast Photoacoustic Tomography. Sci Rep. 2016;6(1):21798. doi: 10.1038/srep21798.

25.	Jenny CH. The mouth and maltreatment: safeguarding issues in child dental health. Archives of Disease in Childhood. 2018;103(8):722. doi: 10.1136/archdischild-2017-313173.

26.	Selwitz RH, Ismail AI, Pitts NB. Dental caries. Lancet. 2007;369(9555):51-9. doi: 10.1016/s0140-6736(07)60031-2. PubMed PMID: 17208642.

27.	Vacaru R-P, Didilescu AC, Constantinescu I, Mărunțelu I, Tănase M, Stanciu IA, et al. Salivary Enzymatic Activity and Carious Experience in Children: A Cross-Sectional Study. Children. 2022;9(3):343. PubMed PMID: doi:10.3390/children9030343.

28.	Pitts NB, Zero DT, Marsh PD, Ekstrand K, Weintraub JA, Ramos-Gomez F, et al. Dental caries. Nat Rev Dis Primers. 2017;3:17030. Epub 20170525. doi: 10.1038/nrdp.2017.30. PubMed PMID: 28540937.

29.	Sabharwal A, Stellrecht E, Scannapieco FA. Associations between dental caries and systemic diseases: a scoping review. BMC Oral Health. 2021;21(1):472. doi: 10.1186/s12903-021-01803-w.

30.	Chen X, Daliri EB, Kim N, Kim JR, Yoo D, Oh DH. Microbial Etiology and Prevention of Dental Caries: Exploiting Natural Products to Inhibit Cariogenic Biofilms. Pathogens. 2020;9(7). Epub 20200714. doi: 10.3390/pathogens9070569. PubMed PMID: 32674310; PubMed Central PMCID: PMCPMC7400585.

31.	Nishi M, Harding M, Kelleher V, Whelton H, Allen F. Knowledge of caries risk factors/indicators among Japanese and Irish adult patients with different socio-economic profiles: a cross-sectional study. BMC Oral Health. 2017;17(1):55. Epub 20170216. doi: 10.1186/s12903-017-0345-x. PubMed PMID: 28209191; PubMed Central PMCID: PMCPMC5314636.

32.	Alotaibi RN, Howe BJ, Chernus JM, Mukhopadhyay N, Sanchez C, Deleyiannis FWB, et al. Genome-Wide Association Study (GWAS) of dental caries in diverse populations. BMC Oral Health. 2021;21(1):377. doi: 10.1186/s12903-021-01670-5.

33.	Ritter AV. Sturdevant's Art & Science of Operative Dentistry - E-Book: Sturdevant's Art & Science of Operative Dentistry - E-Book: Elsevier Health Sciences; 2017.

34.	Tinanoff N, Palmer CA. Dietary Determinants of Dental Caries and Dietary Recommendations for Preschool Children. J Public Health Dent. 2000;60(3):197-206. doi: <https://doi.org/10.1111/j.1752-7325.2000.tb03328.x>.

35.	Murali P, Venkatasubramanian V, Ram B. Sugar: Myths and Reality. In: Mohan N, Singh P, editors. Sugar and Sugar Derivatives: Changing Consumer Preferences. Singapore: Springer Singapore; 2020. p. 253-61.

36.	Bowen WH, Amsbaugh SM, Monell-Torrens S, Brunelle J. Effects of varying intervals between meals on dental caries in rats. Caries research. 1983;17(5):466-71. doi: 10.1159/000260703. PubMed PMID: 6577963.

37.	Doichinova L, Bakardjiev P, Peneva M. Assessment of food habits in children aged 6–12 years and the risk of caries. Biotechnology & Biotechnological Equipment. 2015;29(1):200-4. doi: 10.1080/13102818.2014.989180.

38.	Liu H-Y, Huang S-T, Hsuao S-Y, Chen C-C, Hu W-C, Yen Y-Y. Dental caries associated with dietary and toothbrushing habits of 6- to 12-year-old mentally retarded children in Taiwan. Journal of Dental Sciences. 2009;4(2):61-74. doi: <https://doi.org/10.1016/S1991-7902(09)60010-6>.

39.	Ma W-Y, Yang C-Y, Shih S-R, Hsieh H-J, Hung CS, Chiu F-C, et al. Measurement of Waist Circumference: midabdominal or iliac crest? Diabetes care. 2013;36(6):1660-6. Epub 12/28. doi: 10.2337/dc12-1452. PubMed PMID: 23275359.

40.	Shi W, Neubeck L, Gallagher R. Measurement matters: A systematic review of waist measurement sites for determining central adiposity. Collegian. 2017;24(5):513-23. doi: <https://doi.org/10.1016/j.colegn.2016.08.009>.

41.	Keys A, Fidanza F, Karvonen MJ, Kimura N, Taylor HL. Indices of relative weight and obesity. Journal of Chronic Diseases. 1972;25(6):329-43. doi: <https://doi.org/10.1016/0021-9681(72)90027-6>.

42.	Shulman JD, Cappelli DP. Chapter 1 - Epidemiology of Dental Caries. In: Cappelli DP, Mobley CC, editors. Prevention in Clinical Oral Health Care. Saint Louis: Mosby; 2008. p. 2-13.

43.	Organization WH. Oral Health Surveys: Basic Methods: World Health Organization; 2013.

44.	Silness J, Löe H. Periodontal Disease in Pregnancy II. Correlation Between Oral Hygiene and Periodontal Condition. Acta odontologica Scandinavica. 1964;22(1):121-35. doi: 10.3109/00016356408993968.

45.	Adham MM, El Kashlan MK, Abdelaziz WE, Rashad AS. The impact of minimally invasive restorative techniques on perception of dental pain among pregnant women: a randomized controlled clinical trial. BMC Oral Health. 2021;21(1):76. doi: 10.1186/s12903-021-01432-3.

46.	Löe H. The Gingival Index, the Plaque Index and the Retention Index Systems. J Periodontol. 1967;38(6):Suppl:610-6. Epub 1967/11/01. doi: 10.1902/jop.1967.38.6.610. PubMed PMID: 5237684.

47.	Cogulu D, Sabah E, Kutukculer N, Ozkinay F. Evaluation of the relationship between caries indices and salivary secretory IgA, salivary pH, buffering capacity and flow rate in children with Down's syndrome. Archives of Oral Biology. 2006;51(1):23-8. doi: <https://doi.org/10.1016/j.archoralbio.2005.06.001>.

48.	Franks EM, Jeltema M, Luck PJ, Beckley J, Foegeding EA, Vinyard CJ. Morphological and masticatory performance variation of mouth behavior groups. Journal of Texture Studies. 2020;51(2):343-51. doi: 10.1111/jtxs.12483.

49.	Kim S, Vickers Z. Liking of food textures and its relationship with oral physiological parameters and mouth-behavior groups. Journal of Texture Studies. 2020;51(3):412-25. doi: 10.1111/jtxs.12504.

50.	Jeong S, Lee J. Effects of cultural background on consumer perception and acceptability of foods and drinks: a review of latest cross-cultural studies. Current Opinion in Food Science. 2021;42:248-56. doi: <https://doi.org/10.1016/j.cofs.2021.07.004>.

51.	Woda A, Foster K, Mishellany A, Peyron MA. Adaptation of healthy mastication to factors pertaining to the individual or to the food. Physiology & behavior. 2006;89(1):28-35. doi: <https://doi.org/10.1016/j.physbeh.2006.02.013>.

52.	Laguna L, Hetherington MM, Chen J, Artigas G, Sarkar A. Measuring eating capability, liking and difficulty perception of older adults: A textural consideration. Food Quality and Preference. 2016;53:47-56. doi: <https://doi.org/10.1016/j.foodqual.2016.05.013>.

53.	Ketel EC, Zhang Y, Jia J, Wang X, de Wijk RA, Chen J, Stieger M. Comparison of and relationships between oral physiology, anatomy and food oral processing behavior of Chinese (Asian) and Dutch (Caucasian) consumers differing in age. Physiology & behavior. 2021;232:113284. doi: <https://doi.org/10.1016/j.physbeh.2020.113284>.

54.	Godlewski AE, Veyrune JL, Nicolas E, Ciangura CA, Chaussain CC, Czernichow S, et al. Effect of Dental Status on Changes in Mastication in Patients with Obesity following Bariatric Surgery. PLOS ONE. 2011;6(7):e22324. doi: 10.1371/journal.pone.0022324.

55.	Hamasha AA, Safadi RA. Coronal caries experience in dentate Jordanian adults. Community dental health. 2008;25(1):50-4. PubMed PMID: 18435235.

56.	Al Qaralleh AS. Jordan and Syrian humanitarian refugees' dilemma: international law perspective. Heliyon. 2022;8(5):e09377. Epub 20220504. doi: 10.1016/j.heliyon.2022.e09377. PubMed PMID: 35574201; PubMed Central PMCID: PMCPMC9096676.

57.	Al-Hadi Hamasha A, Sasa I, Al Qudah M. Risk indicators associated with tooth loss in Jordanian adults. Community dentistry and oral epidemiology. 2000;28(1):67-72. doi: <https://doi.org/10.1034/j.1600-0528.2000.280109.x>.

58.	Roberts‐Thomson KF, Luzzi L, Brennan DS. Social inequality in use of dental services: relief of pain and extractions. Australian and New Zealand Journal of Public Health. 2008;32(5):444-9. doi: <https://doi.org/10.1111/j.1753-6405.2008.00277.x>.

59.	Namal N, Can G, Vehid S, Koksal S, Kaypmaz A. Dental health status and risk factors for dental caries in adults in Istanbul, Turkey. EMHJ-Eastern Mediterranean Health Journal, 14 (1), 110-118, 2008. 2008.

60.	Wilson A, Jeltema M, Morgenstern MP, Motoi L, Kim E, Hedderley D. Comparison of physical chewing measures to consumer typed Mouth Behavior. Journal of Texture Studies. 2018;49(3):262-73. doi: <https://doi.org/10.1111/jtxs.12328>.

61.	Kim S. Liking of food textures and relationship with oral physiologicalparameters. Minneapolis, MN: University of Minnesota; 2016.

62.	Birkeland JM, Jorkjend L. The effect of chewing apples on dental plaque and food debris. Community dentistry and oral epidemiology. 1974;2(4):161-2. doi: 10.1111/j.1600-0528.1974.tb01867.x. PubMed PMID: 4530764.

63.	Schneider HG, Knieknecht I. [Reduction of dental plaque by apples and chewing gum]. Nahrung. 1986;30(9):907-12. PubMed PMID: 3796719.

64.	Rubido S, García-Caballero L, Abeleira MT, Limeres J, García M, Diz P. Effect of chewing an apple on dental plaque removal and on salivary bacterial viability. PLOS ONE. 2018;13(7):e0199812. doi: 10.1371/journal.pone.0199812.

65.	Castro ALS, Vianna MIP, Mendes CMC. Comparison of caries lesion detection methods in epidemiological surveys: CAST, ICDAS and DMF. BMC Oral Health. 2018;18(1):122. doi: 10.1186/s12903-018-0583-6.

66.	Castro ALS, Vianna MIP, Mendes CMC. The knowledge and use of population-based methods for caries detection. BMC Oral Health. 2018;18(1):153. doi: 10.1186/s12903-018-0612-5.

67.	Åkesson M-L, Wärnberg Gerdin E, Söderström U, Lindahl B, Johansson I. Health-related quality of life and prospective caries development. BMC Oral Health. 2016;16(1):15. doi: 10.1186/s12903-016-0166-3.

68.	Peres KG, Peres MA, Antunes JLF. Dental Caries. In: Peres MA, Antunes JLF, Watt RG, editors. Oral Epidemiology: A Textbook on Oral Health Conditions, Research Topics and Methods. Cham: Springer International Publishing; 2021. p. 39-56.

69.	Shen A, Bernabé E, Sabbah W. The bidirectional relationship between weight, height and dental caries among preschool children in China. PLOS ONE. 2019;14(4):e0216227. doi: 10.1371/journal.pone.0216227.

70.	Vergnes J-N, Kaminski M, Lelong N, Musset A-M, Sixou M, Nabet C, for the Eg. Frequency and Risk Indicators of Tooth Decay among Pregnant Women in France: A Cross-Sectional Analysis. PLOS ONE. 2012;7(5):e33296. doi: 10.1371/journal.pone.0033296.

71.	Iwasaki M, Kakuta S, Ansai T. Associations among internet addiction, lifestyle behaviors, and dental caries among high school students in Southwest Japan. Sci Rep. 2022;12(1):17342. doi: 10.1038/s41598-022-22364-0.

72.	Olczak-Kowalczyk D, Krämer N, Gozdowski D, Turska-Szybka A. Developmental enamel defects and their relationship with caries in adolescents aged 18 years. Sci Rep. 2023;13(1):4932. doi: 10.1038/s41598-023-31717-2.

73.	Nascimento GG, Chaffee BW. Causal Inference in Oral Health Epidemiology. In: Peres MA, Antunes JLF, Watt RG, editors. Oral Epidemiology: A Textbook on Oral Health Conditions, Research Topics and Methods. Cham: Springer International Publishing; 2021. p. 267-78.

74.	Campus G, Cagetti MG, Senna A, Blasi G, Mascolo A, Demarchi P, Strohmenger L. Does Smoking Increase Risk for Caries? A Cross-Sectional Study in an Italian Military Academy. Caries research. 2011;45(1):40-6. doi: 10.1159/000322852.

75.	Peng SM, Wong HM, King NM, McGrath C. Association between Dental Caries and Adiposity Status (General, Central, and Peripheral Adiposity) in 12-Year-Old Children. Caries research. 2013;48(1):32-8. doi: 10.1159/000351690.

76.	Albahrani MM, Alyahya A, Qudeimat MA, Toumba KJ. Salivary fluoride concentration following toothbrushing with and without rinsing: a randomised controlled trial. BMC Oral Health. 2022;22(1):53. doi: 10.1186/s12903-022-02086-5.

77.	Creeth J, Zero D, Mau M, Bosma ML, Butler A. The effect of dentifrice quantity and toothbrushing behaviour on oral delivery and retention of fluoride in vivo. International Dental Journal. 2013;63:14-24. doi: <https://doi.org/10.1111/idj.12075>.

78.	Hawley GM, Holloway PJ, Davies RM. Dental health status associated with documented dental attendance patterns in adolescents. Community dental health. 1997;14(1):22-4. PubMed PMID: 9114545.

79.	Lopez R, Baelum V. Factors associated with dental attendance among adolescents in Santiago, Chile. BMC Oral Health. 2007;7(1):4. doi: 10.1186/1472-6831-7-4.

80.	Wendell S, Wang X, Brown M, Cooper ME, DeSensi RS, Weyant RJ, et al. Taste Genes Associated with Dental Caries. Journal of Dental Research. 2010;89(11):1198-202. doi: 10.1177/0022034510381502.

81.	Wang X, Shaffer JR, Weyant RJ, Cuenco KT, DeSensi RS, Crout R, et al. Genes and their effects on dental caries may differ between primary and permanent dentitions. Caries research. 2010;44(3):277-84. Epub 20100527. doi: 10.1159/000314676. PubMed PMID: 20516689; PubMed Central PMCID: PMCPMC2919434.

82.	Chisini LA, Varella de Carvalho R, dos Santos Costa F, Salvi LC, Demarco FF, Britto Correa M. Genes and single nucleotide polymorphisms in the pathway of saliva and dental caries: a systematic review and meta-analysis. Biofouling. 2023;39(1):8-23. doi: 10.1080/08927014.2022.2162891.

83.	Gao X, Jiang S, Koh D, Hsu C-YS. Salivary biomarkers for dental caries. Periodontology 2000. 2016;70(1):128-41. doi: <https://doi.org/10.1111/prd.12100>.

84.	Cunha-Cruz J, Scott J, Rothen M, Mancl L, Lawhorn T, Brossel K, Berg J. Salivary characteristics and dental caries: Evidence from general dental practices. The Journal of the American Dental Association. 2013;144(5):e31-e40. doi: <https://doi.org/10.14219/jada.archive.2013.0159>.

85.	Chen Q, Jin X, Zhu H, Salehi HS, Wei K. 3D distribution of dental plaque on occlusal surface using 2D-fluorescence-image to 3D-surface registration. Computers in Biology and Medicine. 2020;123:103860. doi: <https://doi.org/10.1016/j.compbiomed.2020.103860>.

86.	Abbass MMS, AbuBakr N, Radwan IA, Rady D, El Moshy S, Ramadan M, et al. The potential impact of age, gender, body mass index, socioeconomic status and dietary habits on the prevalence of dental caries among Egyptian adults: a cross-sectional study. F1000Res. 2019;8:243. Epub 20190301. doi: 10.12688/f1000research.17892.1. PubMed PMID: 30906540; PubMed Central PMCID: PMCPMC6426081.

87.	Martinez-Mier EA, Zandona AF. The Impact of Gender on Caries Prevalence and Risk Assessment. Dental Clinics of North America. 2013;57(2):301-15. doi: <https://doi.org/10.1016/j.cden.2013.01.001>.

88.	Shaffer JR, Wang X, McNeil DW, Weyant RJ, Crout R, Marazita ML. Genetic Susceptibility to Dental Caries Differs between the Sexes: A Family-Based Study. Caries research. 2015;49(2):133-40. doi: 10.1159/000369103.

89.	Griffin SO, Jones JA, Brunson D, Griffin PM, Bailey WD. Burden of Oral Disease Among Older Adults and Implications for Public Health Priorities. American Journal of Public Health. 2012;102(3):411-8. doi: 10.2105/ajph.2011.300362. PubMed PMID: 22390504.

90.	Oscarson N, Espelid I, Jönsson B. Is caries equally distributed in adults? A population-based cross-sectional study in Norway - the TOHNN-study. Acta odontologica Scandinavica. 2017;75(8):557-63. Epub 20170728. doi: 10.1080/00016357.2017.1357080. PubMed PMID: 28754061.

91.	Edman K, Öhrn K, Nordström B, Holmlund A. Prevalence of dental caries and influencing factors, time trends over a 30-year period in an adult population. Epidemiological studies between 1983 and 2013 in the county of Dalarna, Sweden. Acta odontologica Scandinavica. 2016;74(5):385-92. Epub 20160524. doi: 10.3109/00016357.2016.1163733. PubMed PMID: 27215270.

92.	Bongo A-KS, Brustad M, Jönsson B. Caries experience among adults in core Sámi areas of Northern Norway. Community dentistry and oral epidemiology. 2021;49(5):401-9. doi: <https://doi.org/10.1111/cdoe.12613>.

93.	Jiang X, Jiang X, Wang Y, Huang R. Correlation between tobacco smoking and dental caries: A systematic review and meta-analysis. Tob Induc Dis. 2019;17:34. Epub 20190419. doi: 10.18332/tid/106117. PubMed PMID: 31516477; PubMed Central PMCID: PMCPMC6662788.

94.	Nordenram A, Hultin M, Kjellman O, Ramström G. Indications for surgical removal of the mandibular third molar. Study of 2,630 cases. Swedish dental journal. 1987;11(1-2):23-9. PubMed PMID: 3473707.

95.	Jung YH, Cho BH. Prevalence of missing and impacted third molars in adults aged 25 years and above. Imaging Sci Dent. 2013;43(4):219-25. Epub 20131212. doi: 10.5624/isd.2013.43.4.219. PubMed PMID: 24380060; PubMed Central PMCID: PMCPMC3873309.

96.	Derossi A, Husain A, Caporizzi R, Severini C. Manufacturing personalized food for people uniqueness. An overview from traditional to emerging technologies. Crit Rev Food Sci Nutr. 2020;60(7):1141-59. Epub 20190122. doi: 10.1080/10408398.2018.1559796. PubMed PMID: 30668142.
