# Supplementary material for: Oral processing behavior and dental caries; an insight into a new relationship
Source: PLoS One. 2024 Jul 2;19(7):e0306143. doi: 10.1371/journal.pone.0306143 (PMC11218957; doi:10.1371/journal.pone.0306143)
Supplement: Attachment — Submitted filename: Response to Reviewers.docx [file pone.0306143.s006.docx]

Melanie Alazzam

Corresponding Author

Assistant Professor

Department of Oral Medicine and Oral Surgery

Faculty of Dentistry

Jordan University of Science and Technology

P.O. Box: 3030, Irbid 22110, Jordan

Telephone: (+962)2-7201000 Ext: 23558

Email address: mfalazam@just.edu.jo

Dr. Hadi Ghasemi

Academic Editor

PLOS ONE

4.30.2024

Dear Dr. Ghasemi,

We hope that this version meets our reviewers’ expectations and addresses their comments. Kindly note that the study is providing an insight into a new potential relationship between oral processing behavior and dental caries. A relationship in which oral processing behavior can affect and/or be affected by an individual’s caries experience. The findings of our study represent a potential area for future research in the field of food oral processing and /or the dental caries process. We kindly ask our reviewers to read the manuscript in light of the study aims, taking into consideration the sample size and the number of confounding factors that were adjusted for in our study. Understanding the multifactorial nature of dental caries guided us into a more humble conclusion and further encouraged us to further investigate this relationship taking into consideration other important confounders that our reviewers have referred to.

In this revision, we made minor changes to elaborate on a few limitations in our study. We have also rewritten a paragraph in the discussion section.

Thank you for taking the time and effort to help us improve this interesting work.

Looking forward to hearing from you!

Sincerely,

Melanie Alazzam

| **Reviewer** | **Comment Number** | **Reviewer’s Comment** | **Authors’ Reply** | **Author’s Action** |
| --- | --- | --- | --- | --- |
| Reviewer (2) | 1 | The manuscript has now been revised and is much improved.  I still think the manuscript is very interesting, and it provides new insights regarding oral health related to oral processing behaviors | We thank you for your interest, feedback, and your valuable comments which helped modify the tone of our study. |  |
| Reviewer (2) | 2 | I still consider the largest limitation in this manuscript to be that the fluoride aspect has not been accounted for in the method. However, this has now been addressed in the discussion. I am still concerned that excluding this factor might have provided a large bias in the study. | We value your concern. We acknowledge this as a limitation in our study. We further elaborated on the statement to highlight that this is a main limitation that may lead to an unwanted bias. | **Line 475 (highlighted in yellow)**  **(Line numbers to be viewed while track changes mode is on)** |
| Reviewer (2) | 3 | Additionally, saliva secretion rates, and buffer capacity has not been included either which is a limitation, since this greatly affects the caries risk. I have commented on this previously. Saliva flow rates related to oral processing behaviors this is briefly discussed in the discussion, acknowledging that there might be differences between the studied groups, but authors should recognize that the neglect of measuring this factor in the current study is as a limitation. | We appreciate this comment. Saliva is an important factor in the caries process and its role should not be marginalized taking into consideration the available evidence. We have pointed to this limitation in our manuscript and elaborated that this limitation may have led to an unwanted bias. | **Line 482 (highlighted in yellow)**  **(Line numbers to be viewed while track changes mode is on)** |
| Reviewer (2) | 4 | In the discussion, (line 446 and forwards) reference 13 is used. This paragraph is confusing. The study by Liu et al 2021 does not support the statement that SPL demonstrated generally lower sweet taste perceptions compared to FPL. In the two groups of the study, Danish and Chinese test persons were compared. For Chinese individuals, the FPL:s demonstrated lower sweet taste perception compared to SPL:s, but the results were the opposite for Danish test persons. The relationship between oral processing behaviour and sweet taste perception is therefore, at least based on the referred study, not clear. I think this paragraph must be rewritten. | Thank you for pointing this out. We have rewritten the paragraph and elaborated on the fact the relationship between sweet taste perception and the preferred oral processing behavior is still not clear but warrants further research. | **Line 432 (highlighted in yellow)**  **(Line numbers to be viewed while track changes mode is on)** |
| Reviewer (3) | 1 | It is unclear where the modification have taken place. The authors response does not detail that. Kindly respond to each question in the authors response letter stating where the modifications happened in the manuscript. | We apologize for this. Our manuscript was reviewed by 6 different reviewers in two revisions. We believe that our response to your comments was addressed in another older version of the response to reviewers (the response dated 12.26.24). **Therefore, below we provided our older response and we have modified the line numbers to the latest version of the manuscript (current manuscript)**. We hope that all your comments have been addressed in this version. | **Kindly see pages (5-9) of this document.**  **(Line numbers to be viewed while track changes mode is on)** |
| Reviewer (4) | 3 | Still more recent studies should be mentioned and more paragraphs should be added to the introduction and discussion with other relevant studies | Dear Prof. Dr. Aldulaimi, we value your recommendations.  Regarding adding more recent studies, we updated the reference list to the best of our ability without affecting the integrity of our manuscript. Kindly note that only 28 references out of 112 references were older than 10 years.  Regarding papers related to preferred oral processing behaviors (mouth behaviors), we have included all relevant papers in our study as far as we know. This topic is new and has just emerged in 2014.    Regarding the discussion section, we have added two paragraphs per your request. The paragraphs discussed two important points. The first point was related to the independence of the oral processing behavior from age and sex. The second point discussed a new finding related to oral processing behavior which is sweet taste perception. A finding that may guide future research into a new area in the study of this potential relationship. | **Line 376 (highlighted in yellow)**  **Line 432 (highlighted in yellow)**  **(Line numbers to be viewed while track changes mode is on)** |

**The First Version of the Response to Reviewers Dated (12.26.24)**

Melanie Alazzam

Corresponding Author

Assistant Professor

Department of Oral Medicine and Oral Surgery

Faculty of Dentistry

Jordan University of Science and Technology

P.O. Box: 3030, Irbid 22110, Jordan

Telephone: (+962)2-7201000 Ext: 23558

Email address: mfalazam@just.edu.jo

Dr. Hadi Ghasemi

Academic Editor

PLOS ONE

22.2.2024

Dear Dr. Ghasemi,

We would like to thank our reviewers for taking the time and effort to review this work. Their feedback is highly appreciated, and it has truly contributed to improving the quality of our manuscript. In what follows, we addressed each comment raised. In general, we made some modifications to the tone of the manuscript to ensure that our discussion and conclusions accurately reflect the findings of the study, while taking into consideration the limitations of our research.

Looking forward to hearing from you!

Sincerely,

Melanie Alazzam

| **Reviewer** | **Comment Number** | **Reviewer’s Comment** | **Authors’ Reply** | **Author’s Action**  **(Line numbers to be viewed while track changes mode is on)** |
| --- | --- | --- | --- | --- |
| Reviewer (1) | 1 | Please include the POPB questionnaire | We appreciate your feedback. We have included the POPB questionnaire per your request. We sought permission from Elsevier to reuse the questionnaire and the translated version in our future publication. The license number is 5662390874243. We did not include the questionnaire previously due to uncertainty about the terms of the permission granted to us. Further correspondence with Elsevier clearly stated that we can reuse and include the POPB questionnaire, and the Arabic version as shown in the uploaded supplementary file. | **A supplementary data file was uploaded “S1 file”** |
| Reviewer (1) | 2 | Using a translated questionnaire for study requires standardization of the translated version (translation, back translation, expert opinion...). Please explain more if you have standardized the question. | Thank you for highlighting this point. The questionnaire was forward translated into the Arabic language by a committee of three competent bilingual speakers. The committee included a linguist (Dr. Aziz Jaber) and two clinicians, Prof. Suhad Aljundi and Dr. Mohammad Atieh. A training session was conducted in which research assistants were introduced to the aims of the study and the POPB questionnaire. Next, the questionnaire was distributed to a pilot sample of 23 participants. Each participant filled out the questionnaire, and comments or questions were recorded by the accompanying research assistant. Another meeting with the research assistants was held to address the recorded comments/feedback regarding the Arabic version of the questionnaire. Based on responses from the pilot sample and the revision done by two of the committee members, two text questions and three photo-based questions were slightly modified. The modifications aimed to include/remove food items that were more familiar/unfamiliar to our study group (details are included in the supplementary data file)  Although back translation is commonly used in the literature as a tool to ensure the accuracy of the translated questionnaire, its use and efficacy as a standard assessment method are controversial. Some researchers have noted inconsistency in the detection of presumed flaws when more individuals are employed to compare back translation with the original text. While back translation can uncover problems, it also leads to a significant number of false alarms. Moreover, a significant number of issues persist, as demonstrated by Behr [1] and Colina [2]. Other researchers argue that using back translation as a quality assessment tool in multilingual survey research is now deemed obsolete or on its way to becoming so, although it is still widely practiced. It is argued that back translation may still be useful as a documentation tool, not as a quality control tool[3].  **Regarding the questionnaire used in the study, most of its items consist of repetitive sentences with food item variables. The food items in English have unique equivalent terms in Arabic, rendering back translation less effective. Instead, we chose to standardize the translated questionnaire using a more accuracy-ensuring method**. | **Materials and methods/ Line 189**:  **“and the experts’ opinion” as this was done but was not clearly stated in the first draft of the manuscript.**  (highlighted in green) |
| Reviewer (3) | 1 | Would it have been meaningful to include patients with minimal missing teeth or possible none to eliminate this confounding factor? | Thank you for highlighting this point. We agree with you. Studying this in a sample of dentate individuals will improve our understanding of this relationship. It will also help us overcome the potential unwanted overestimation of the “M” component due to the different biases discussed in the manuscript. Accordingly, this point was added to the limitations of the study. | **Discussion / Line 463 & 516**  **This was added as a new limitation to our research and to our conclusions.**  (highlighted in light blue) |
| Reviewer (3) | 2 | With the information you have collected would it not have been more meaningful to perform caries risk assessment? I encourage you to present it in caries risk format if you are able to. It would simplify data presentation and make comparisons with literature easier since most studies are reporting in that way. | Thank you for the suggestion. We agree with your point. However, doing this retrospectively seemed to be unfeasible since certain factors in the available caries risk assessment forms/tools were not included in our questionnaire. The ADA caries risk assessment form, Cariogram, AAPD CRA Tool, and CAries Management By Risk Assessment (CAMBRA) are an example. Nevertheless, we are planning to use the caries assessment form/tools in the near future to highlight and understand the relationship between oral processing and dental caries. | -------- |
| Reviewer (3) | 3 | What is the importance of collecting plaque indices since its relationship with dental caries has proven to be weak | We appreciate asking about this point. When conducting this study, we had the hypothesis that differences in the preferred oral processing behavior are potentially associated with differences in dental plaque levels. Based on the available evidence**[4-7]**, we assumed that differences in dental plaque levels may contribute to differences in caries experience. Accordingly, we anticipated that FPL, who preferred using their teeth during oral processing and preferred hard food textures would demonstrate lower plaque levels compared to SPL. This hypothesis was derived from a study demonstrating changes in dental plaque levels when chewing an apple**[8, 9]**. A food texture that is most likely not preferred by SPL group **[10]**.  Your feedback (comments 1 and 3) were helpful in changing the tone of our study. | **Discussion / Line: 420**  **The purpose of collecting dental plaque and its related results were**  **added and discussed per your feedback.**  (highlighted in light blue) |

**References:**

1.	Behr D. Assessing the use of back translation: the shortcomings of back translation as a quality testing method. International Journal of Social Research Methodology. 2017;20(6):573-84. doi: 10.1080/13645579.2016.1252188.

2.	Colina S, Marrone N, Ingram M, Sánchez D. Translation Quality Assessment in Health Research: A Functionalist Alternative to Back-Translation. Evaluation & the Health Professions. 2017;40(3):267-93. doi: 10.1177/0163278716648191. PubMed PMID: 27207929.

3.	Son J. Back translation as a documentation tool. Translation & Interpreting: The International Journal of Translation and Interpreting Research. 2018;10(2):89–100.

4.	Laajala A, Pesonen P, Anttonen V, Laitala M-L. Association of Enamel Caries Lesions with Oral Hygiene and DMFT among Adults. Caries research. 2019;53(4):475-81. doi: 10.1159/000497358.

5.	Leroy R, Bogaerts K, Martens L, Declerck D. Risk factors for caries incidence in a cohort of Flemish preschool children. Clin Oral Investig. 2012;16(3):805-12. doi: 10.1007/s00784-011-0579-y.

6.	Warren JJ, Weber-Gasparoni K, Marshall TA, Drake DR, Dehkordi-Vakil F, Kolker JL, Dawson DV. Factors Associated with Dental Caries Experience in 1-Year-Old Children. J Public Health Dent. 2008;68(2):70-5. doi: <https://doi.org/10.1111/j.1752-7325.2007.00068.x>.

7.	Declerck D, Leroy R, Martens L, Lesaffre E, Garcia-Zattera M-J, Broucke SV, et al. Factors associated with prevalence and severity of caries experience in preschool children. Community dentistry and oral epidemiology. 2008;36(2):168-78. doi: <https://doi.org/10.1111/j.1600-0528.2007.00385.x>.

8.	Schneider HG, Knieknecht I. [Reduction of dental plaque by apples and chewing gum]. Nahrung. 1986;30(9):907-12. PubMed PMID: 3796719.

9.	Rubido S, García-Caballero L, Abeleira MT, Limeres J, García M, Diz P. Effect of chewing an apple on dental plaque removal and on salivary bacterial viability. PLOS ONE. 2018;13(7):e0199812. doi: 10.1371/journal.pone.0199812.

10.	Jeltema M, Beckley J, Vahalik J. Food texture assessment and preference based on Mouth Behavior. Food Quality and Preference. 2016;52:160-71. doi: <https://doi.org/10.1016/j.foodqual.2016.04.010>.
