# Supplementary material for: Oral processing behavior and dental caries; an insight into a new relationship
Source: PLoS One. 2024 Jul 2;19(7):e0306143. doi: 10.1371/journal.pone.0306143 (PMC11218957; doi:10.1371/journal.pone.0306143)
Supplement: Attachment — Submitted filename: Response to Reviewers.docx [file pone.0306143.s007.docx]

Melanie Alazzam

Corresponding Author

Assistant Professor

Department of Oral Medicine and Oral Surgery

Faculty of Dentistry

Jordan University of Science and Technology

P.O. Box: 3030, Irbid 22110, Jordan

Telephone: (+962)2-7201000 Ext: 23558

Email address: mfalazam@just.edu.jo

Dr. Hadi Ghasemi

Academic Editor

PLOS ONE

5.28.2024

Dear Dr. Ghasemi,

We thank you and the reviewers for your continued support and interest in our manuscript. We would like to confirm that in all 4 revisions of this manuscript, we provided a “response to reviewers” file. The file in each of the 4 revisions contained a cover letter and a table that included 5 columns (Reviewer number, Comment number, Reviewer’s comment, Authors’ reply, Author’s action). The cover letter and the table were in landscape style. We are concerned that for a technical reason, the “response to reviewers” file did not appear for the reviewers last time. We remember encountering a note at PLOS One editorial system which indicated that there were some technical issues with the website. We deeply appreciate the effort our reviewers put into this review process and believe that not addressing each comment is disrespectful to our colleague researchers. Therefore, we were/are always very keen to address each and every comment received.

Below, we provide our response to the last revision we received. In addition, we provide our response to the comments that we expect were provided by reviewers “2” and “3” in previous revisions as well.

Please note that the comments were organized in a table as we have just described above. We highlighted the comments provided by reviewer “2” in light blue and the comments provided by reviewer “3” in yellow.

We thank you for your understanding and we look forward to hearing from you!

Sincerely,

Melanie Alazzam

| **Reviewer** | **Comment Number** | **Reviewer’s Comment** | **Authors’ Reply** | **Author’s Action** |
| --- | --- | --- | --- | --- |
| Reviewer (2) | 1 | I have again reviewed the manuscript. As a whole, I think the manuscript now is suitable for publication. However, I have a few comments. | We thank you for your interest, feedback, and your valuable comments which helped modify the tone of our study. |  |
| Reviewer (2) | 2 | In the discussion, there is a minor language error. Line 422: “Despite being controversial [68], hard food (including apples) was found to reduce the accumulation levels of dental plaque [69]”. This statement is referring to a study, thus this should be clearly stated : “Despite being controversial [68], a study has found that…". | Thank you for your feedback. We have noticed that the paragraph was unclear and indeed indicated what you have pointed out. Therefore, we have rewritten the paragraph to make the paragraph less confusing to the reader. | **Discussion Section**  **(Line 419-441 in Track changes file”)**  The paragraph was rewritten as in the following:  “In addition to assessing dental caries experience, dental plaque levels were evaluated among the different oral processing behaviors among the groups. The dental plaque (biofilm) is known for its role in the development of dental caries and periodontal disease[68, 69]. Despite being controversial[70], previous evidence indicated that certain food textures such as hard food (including apples) were found to reduce dental plaque accumulation levels[71]. Since variations in food texture preference were previously reported between the oral processing (mouth behavior) groups[7, 8, 11], it was of interest in this project to determine dental plaque levels among the different oral processing behaviors. Although plaque accumulation levels were similar between FPL and SPL, a significant difference in dental caries experience was demonstrated. This was in agreement with previous reports in which dental plaque accumulation levels demonstrated a positive but weak relationship with the dental caries experience[72-74]. The findings in this study at least partially indicate This was done to better understand one of the aspects of this potential relationship. Despite being controversial, hard food (including apples) was found to reduce the accumulation levels of dental plaque. Accordingly, it was suggested that variations in food texture preference among the different oral processing behaviors would influence dental plaque levels. However, SPL and FPL demonstrated similar levels of plaque accumulation. A possible interpretation for this was that oral processing behavior might affect an individual’s caries experience by means other than the dental plaque levels. These could be related to variations in biofilm structure and composition[69, 75, 76], different or other salivary parameters such as the salivary bacterial viability[77], and salivary flow rate [52]. Indeed, preliminary data indicates a higher salivary flow rate among crunchers and suckers[52]. Although differences in salivary flow rate were observed, the sample size was small (crunchers n= 41, suckers n= 4)[52].” |
| Reviewer (2) | 3 | In contrary to the authors, I am not surprised by the findings that there was no difference in dental caries prevalence related to plaque levels. Several previous studies have shown that in general, there are only weak correlations between dental caries and dental plaque levels, except from root caries where a stronger correlation can be observed. | We appreciate your guidance. We got your point regarding the weak positive relationship between dental caries and dental plaque accumulation levels. This is helpful in explaining our findings. Therefore, we included this in our discussion. | **Discussion Section**  **(Line 426 in Track changes file”)**  **The following statement was added**:  “Although plaque accumulation levels were similar between FPL and SPL, a significant difference in dental caries experience was demonstrated. This was in agreement with previous reports in which dental plaque accumulation levels demonstrated a positive but weak relationship with the dental caries experience[72-74].” |
| Reviewer (2) | 4 | On line 475, a statement has been included to clarify the bias of not accounting for fluoride in the study. However, I think this statement should be rephrased (referring to the authors themselves in the text as “we” is not proper academic language). | Thank you for your feedback. The statement “The fact that we did not account for fluoride use in our sample represents a main limitation in this study that may have led to an unwanted bias.” was changed into a passive voice. | **Discussion Section**  **(Line 487 in Track changes file”)**  **The statement was changed to** “The fact that fluoride use was not accounted for in this study represents a main limitation that may have led to an unwanted bias.” |
| Reviewer (2) | 5 | As a side note, I must say that I am surprised that the authors have not addressed and responded to the reviewers’ questions one by one, as is expected when resubmitting a revised manuscript. | We apologize for this misunderstanding. We believe there was a technical problem that prevented you from accessing our response, or our response to your comments was evaluated by a second group of reviewers who examined our manuscript. Below (on page =19) we provide our response to your comments dated on 2.22.2024. We hope they address your concerns. | We believe that your comments were addressed in our response dated on **22.2.2024**. Kindly see below on page 19. |
| Reviewer (3) | * | Kindly respond in detail to original comments while making the change clear in the authors response. for example: materials in methods, line 2: change is the so and so | Thank you for bringing up this point. This is a concern for us. We provided a table that included our response to your comments in the “response to reviewers” dated on 4.30.2024. We believe that there might have been a technical problem that prevented you and reviewer “2” from accessing this file. | We provide our response to your comments that were addressed in the “response to reviewer” file dated on **4.30.2024** highlighted in green. Moreover, we provide our response to your comments that were addressed in the “response to reviewers” file dated on **22.2.2024** highlighted in yellow. |
| Reviewer (3) | 1 | The manuscript has now been revised and is much improved.  I still think the manuscript is very interesting, and it provides new insights regarding oral health related to oral processing behaviors | We thank you for your interest, feedback, and your valuable comments which helped modify the tone of our study. |  |
| Reviewer (3) | 2 | I still consider the largest limitation in this manuscript to be that the fluoride aspect has not been accounted for in the method. However, this has now been addressed in the discussion. I am still concerned that excluding this factor might have provided a large bias in the study. | We value your concern. We acknowledge this as a limitation in our study. We further elaborated on the statement to highlight that this is a main limitation that may lead to an unwanted bias. | **Discussion Section**  **(Line 488-489 in Track changes file )**  **The following statement was added**  “The fact that fluoride use was not accounted for in this study represents a main limitation that may have led to an unwanted bias. However, previous reports have shown that the use of fluoridated toothpaste among Jordanians varied between (~62%-92%)” |
| Reviewer (3) | 3 | Additionally, saliva secretion rates, and buffer capacity has not been included either which is a limitation, since this greatly affects the caries risk. I have commented on this previously. Saliva flow rates related to oral processing behaviors this is briefly discussed in the discussion, acknowledging that there might be differences between the studied groups, but authors should recognize that the neglect of measuring this factor in the current study is as a limitation. | We appreciate this comment. Saliva is an important factor in the caries process and its role should not be marginalized taking into consideration the available evidence.  We have pointed out this limitation in our manuscript and elaborated that this limitation may have led to an unwanted bias. | **Discussion Section**  **(Line 495-497 in Track changes file)**  **The following statement was added** “The lack of recording these salivary parameters may have been a possible source of bias and a limitation that needs further attention when interpreting the findings of this study. ” |
| Reviewer (3) | 4 | In the discussion, (line 446 and forwards) reference 13 is used. This paragraph is confusing. The study by Liu et al 2021 does not support the statement that SPL demonstrated generally lower sweet taste perceptions compared to FPL. In the two groups of the study, Danish and Chinese test persons were compared. For Chinese individuals, the FPL:s demonstrated lower sweet taste perception compared to SPL:s, but the results were the opposite for Danish test persons. The relationship between oral processing behavior and sweet taste perception is therefore, at least based on the referred study, not clear. I think this paragraph must be rewritten. | Thank you for pointing this out. We have rewritten the paragraph as you have suggested in your comment. And elaborated on the fact that the relationship is unclear. | **Discussion section**  **(Line 443-458 in Track changes file)**  We rearranged the first part of the paragraph as in the following  **(Line 443 in Track changes file)**  “Besides these suggested means, a recent study demonstrated ethnic variation in sweet taste perception among the different oral processing behaviors[13]. In the two groups of the study, Danish and Chinese participants were compared in terms of sweet taste perception. For the Danish group, SPL demonstrated lower sweet taste perception compared to FPL. They provided a lower rating for the sweetness of different yogurt samples when compared to FPL[13]. The opposite was observed in the Chinese study group[13].  We added the following statement in this paragraph  **(Line 449 in Track changes file)**  “Although the relationship between sweet taste perception and oral processing behaviors is still unclear, further research is warranted as evidence suggests that lower sweet taste perception is associated with greater sucrose intake and increased caries experience[71-73]”  **We rearranged the last part of the paragraph as in the following**  **(Line 452 in Track changes file)**  “The fact that the findings of our study did not demonstrate differences in the consumption of sugary drinks does not contradict the previous evidence. The lack of such difference in sugary drinks consumption between FPL and SPL in our sample could be attributed to the method used to collect the frequency of sugary drinks intake (short food frequency questionnaire) [74]. A more comprehensive way of reporting food intake may reveal such differences between the different oral processing behaviors[75, 76]” |

**The First Version of the Response to Reviewers Dated (22.2.2024)**

Melanie Alazzam

Corresponding Author

Assistant Professor

Department of Oral Medicine and Oral Surgery

Faculty of Dentistry

Jordan University of Science and Technology

P.O. Box: 3030, Irbid 22110, Jordan

Telephone: (+962)2-7201000 Ext: 23558

Email address: mfalazam@just.edu.jo

Dr. Hadi Ghasemi

Academic Editor

PLOS ONE

22.2.2024

Dear Dr. Ghasemi,

We would like to thank our reviewers for taking the time and effort to review this work. Their feedback is highly appreciated, and it has truly contributed to improving the quality of our manuscript. In what follows, we addressed each comment raised. In general, we made some modifications to the tone of the manuscript to ensure that our discussion and conclusions accurately reflect the findings of the study, while taking into consideration the limitations of our research.

Looking forward to hearing from you!

Sincerely,

Melanie Alazzam

| **Reviewer** | **Comment Number** | **Reviewer’s Comment** | **Authors’ Reply** | **Author’s Action** |
| --- | --- | --- | --- | --- |
| Reviewer (1) | 1 | Please include the POPB questionnaire | We appreciate your feedback. We have included the POPB questionnaire per your request. We sought permission from Elsevier to reuse the questionnaire and the translated version in our future publication. The license number is 5662390874243. We did not include the questionnaire previously due to uncertainty about the terms of the permission granted to us. Further correspondence with Elsevier clearly stated that we can reuse and include the POPB questionnaire, and the Arabic version as shown in the uploaded supplementary file. | **A supplementary data file was uploaded “S1 file”** |
| Reviewer (1) | 2 | Using a translated questionnaire for study requires standardization of the translated version (translation, back translation, expert opinion...). Please explain more if you have standardized the question. | Thank you for highlighting this point. The questionnaire was forward translated into the Arabic language by a committee of three competent bilingual speakers. The committee included a linguist (Dr. Aziz Jaber) and two clinicians, Prof. Suhad Aljundi and Dr. Mohammad Atieh. A training session was conducted in which research assistants were introduced to the aims of the study and the POPB questionnaire. Next, the questionnaire was distributed to a pilot sample of 23 participants. Each participant filled out the questionnaire, and comments or questions were recorded by the accompanying research assistant. Another meeting with the research assistants was held to address the recorded comments/feedback regarding the Arabic version of the questionnaire. Based on responses from the pilot sample and the revision done by two of the committee members, two text questions and three photo-based questions were slightly modified. The modifications aimed to include/remove food items that were more familiar/unfamiliar to our study group (details are included in the supplementary data file)  Although back translation is commonly used in the literature as a tool to ensure the accuracy of the translated questionnaire, its use and efficacy as a standard assessment method are controversial. Some researchers have noted inconsistency in the detection of presumed flaws when more individuals are employed to compare back translation with the original text. While back translation can uncover problems, it also leads to a significant number of false alarms. Moreover, a significant number of issues persist, as demonstrated by Behr [1] and Colina [2]. Other researchers argue that using back translation as a quality assessment tool in multilingual survey research is now deemed obsolete or on its way to becoming so, although it is still widely practiced. It is argued that back translation may still be useful as a documentation tool, not as a quality control tool[3].  **Regarding the questionnaire used in the study, most of its items consist of repetitive sentences with food item variables. The food items in English have unique equivalent terms in Arabic, rendering back translation less effective. Instead, we chose to standardize the translated questionnaire using a more accuracy-ensuring method**. | **The following four words were added:**  “and the experts’ opinion” as this was done but was not clearly stated in the first draft of the manuscript. |
| Reviewer (2) | 1 | Initial lesions were not recorded, and radiographic imaging was not used, why? As the authors must know, without radiographs there will be problems with false negative outcomes. I know this is discussed in the discussion, but this is truly a major issue. | Thank you for highlighting this point. We acknowledge the relevance of this matter.  Initial lesions were not recorded as we chose the DMFS index to assess caries experience. The index was chosen since it's time-saving and easy to apply **[4, 5].** When assessing caries experience using the DMFS, the WHO guidelines were followed which exclude early non-cavitated enamel lesions. As we designed the study, we took into consideration that using the DMFS index would underestimate caries experience compared to other methods such as the ICDAS and CAST**[4]**. This would further challenge our hypothesis and would make it harder to detect a difference in caries experience among the study groups.  Radiographs were not taken for financial and participant-related reasons. This research was a non-funded project. Furthermore, we collected many variables including dental caries experience and the plaque index. Recording the plaque index is time-consuming and tiring for the participant. Taking radiographs was compromised to make the data collection session shorter and to reach a larger sample size. Such a compromise was anticipated to underestimate caries experience and would further challenge our hypothesis. The underestimation would make it harder to detect a difference in caries experience among the groups. However, a significant difference in caries experience was detected between the different oral processing behaviors.  Nevertheless, we believe that using the DMFS index with no bitewing radiographs was useful for the purpose of exploring a potential relationship similar to other studies investigating new associations with dental caries**[6-9].** | **Discussion Section**  **(Line 397 in Track changes file)**  **The statement was re-written as in the following:**  “The former observation could be attributed to the underestimation of dental caries as initial lesions were not recorded and radiographs were not taken”  **Discussion Section**  **(Line 466-471 in Track changes file)**  **The following was added as a new limitation to the study**  “The first limitation was related to dental caries assessment. Dental caries was assessed without taking bitewing radiographs. This may have led to an underestimation of dental caries experience in both groups. Moreover, the DMFS index was used to assess dental caries experience. The index is easy to apply and is not time-consuming[65, 66]. However, according to the WHO guidelines initial enamel lesions were not recorded[43, 65] . This may have caused an underestimation of dental caries.” |
| Reviewer (2) | 2 | DMFT was significantly higher in one of the groups, however, when looking at the individual aspects, only the M = missing was significantly higher. Is it a correct conclusion to draw that dental caries has a correlation with eating behaviors in this study? For me, it is rather oral health and not specifically dental caries that can be discussed since decayed and filled were not significantly higher in one of the groups. The limitation of the DMFT-index is that the M = missing part is always unknown regarding the etiology. | We value your concern. Indeed, we had similar concerns about our findings which were included in our discussion. Although we provided an evidence-based explanation for our findings, your feedback (in comments 2,3,4,5) enabled us to gain a deeper understanding of our data and implement modifications that accurately represent the insights we’ve discovered. Therefore, we made several changes in the manuscript that are detailed in the author’s actions column. In general, these amendments included the title, abstract, discussion and conclusions. | **Title Page**  **added** “an insight into” **the title**.  **Abstract**  **(Line 39 in Track changes file)**  **changed the word** “determine” to “explore if a relationship exists” **in the aims of the study.**  **Abstract**  **(Line 47 in Track changes file)**  **deleted the sentence** “The preferred oral processing behavior did not show any predilection for sex(P=0.409) or age(P=0.053).”  **Abstract**  **(Line 50 in Track changes file)**  **deleted the sentence** “ In contrast, the mean total plaque score was similar 0.61(± 0.60) for both groups (P=0.964). Plaque accumulation levels did not vary significantly per quadrant or arch”.  **Abstract**  **(Line 49 in Track changes file)**  **added the sentence** “exhibited a significantly higher mean score for the “M” component (P <0.001) while no significant difference in the mean score of the “D”(P= 0.076), and “F”(P=0.272) components was observed when compared to FPL.”  **The above changes in lines 47,49, 50 were made to display outcomes that were more relevant to our conclusions**.  **Abstract**  **(Line 52 in Track changes file)**  **modified the conclusion for a better representation of the insights discovered as in the following**:    “The current findings provide new insight into a possible relationship between the preferred oral processing behavior and an individual’s caries experience. A relationship in which the preferred oral processing behavior can potentially affect and/or be affected by the dental caries experience.”  **Introduction Section**  **(Line 137 in Track changes file)**  **Amendments were made in the aims section that explain the aims and their boundaries in a more accurate manner as in the following:**  “Therefore, this study aimed to investigate if a relationship exists between the preferred oral processing behavior (FPL vs SPL) and dental caries by examining caries experience among these groups. Although the study does not aim to establish a cause-effect relationship, it aims to shed light on a potential link between the oral processing behavior and dental caries”  **Discussion Section**  **(Line 365 in Track changes file)**  **Confirmation of the actual aims of the study and exclusion of other aims that could be mistakenly perceived by the reader. This was done by writing the following:**  “The second aim was to investigate whether there is a relationship between an individual’s preferred oral processing behavior and their dental caries experience. The study did not intend to explore a cause-effect association but rather to give a new insight into a potential factor that may affect or be affected by dental caries”  **Discussion Section**  **(Line 385 in Track changes file)**  **Adding the word** “possible” **to the sentence** “The third key finding was the existence of a possible relationship between the individual’s preferred oral processing behavior and dental caries experience”    **Discussion Section**  **(Line 386 in Track changes file)**  **Adding the following statement**  “ However, the direction and the nature of such a relationship remain unclear. The preferred oral processing behavior may affect and/ or be affected by dental caries experience.”  **Discussion**  **(Line 394-406 in Track changes file)**  **This part was re-written partly to make sure the paper is not favoring a certain direction for the discovered relationship but rather is encouraging the reader to consider all aspects of such potential association**:  “Nevertheless, there are a few caveats to such interpretation. Although the mean DMFS score varied significantly between the different oral processing behaviors, no difference in the mean decayed “D” or filled “F” components of the DMFS was observed between the groups. The former observation could be attributed to the underestimation of dental caries as initial lesions were not recorded and radiographs were not taken. The latter observation was probably related to both groups having similar patterns to using available dental services[61]. Jordan is perceived as a country with limited resources and facing economic challenges[62]. In poor societies, extraction is sought more frequently than restorative treatment[63-65] which likely played a role in the negligible difference observed in the mean score of the “F” component. Therefore, in this study SPL had a greater number of teeth that possibly needed extraction due to caries compared to FPL. This may have contributed to the significant difference in the mean score of the missing “M” component of the DMFS between the groups.”  **Discussion**  **(Line 471-475 in Track changes file)**  **The limitations of the DMFS , ,specifically the M component, was addressed. We discussed the biases inherent within the “M” component to keep the reader aware of these limitations when examining our data.**  **The following statements were added**:    “Conversely, an overestimation of the “M” component may have been caused by recall bias and other biases pertinent to the calculation of the “M” component in individuals ≥ 30 years[44, 79]. While the score of the “M” component depends on losses due to dental caries, other reasons for tooth loss such as trauma, periodontal disease, and orthodontic reasons, are included in the calculations of the “M” component in this age group.”  **Discussion**  **(Line 523 in Track changes file)**  **Rewritten the conclusion for a better representation of the insights discovered. The first part of the conclusion was written as in the following:**  “In conclusion, an individual’s preferred oral processing behavior could potentially be linked to their caries experience. Although the current findings don’t support a causal association, they provide a new insight into a possible relationship. A relationship in which oral processing behavior can affect and/or be affected by an individual’s caries experience. Future longitudinal studies are needed to confirm this relationship and to understand its nature. Moreover, it is necessary to assess the generalizability of our findings to other ethnic/racial groups.” |
| Reviewer (2) | 3 | In the manuscript, the aspect of seeking dental care is discussed, and how this affects the results. The question of seeking dental care, and specifically asking about why and what kind of care (only emergency, regular check-ups, filling therapy) should have been included in the questionnaire. | We appreciate highlighting this matter. Indeed, evidence suggests that the number of missing teeth may vary depending on the reason for seeking dental care. For instance, there was an association between seeking dental care when having symptoms versus not having symptoms and the number of missing teeth **[10, 11]**. Therefore, we added this as a limitation to our study while keeping in mind the complex nature of dental caries and the difficulty of addressing all confounders in a single study**[12]**. | **Discussion**  **(Line 489 in Track changes file)**  **This was added as a new limitation to our research. The following statements were added**:  “Moreover, the pattern of seeking dental care was evaluated, but no data was gathered about the reasons for seeking dental care and the type of treatment received. Evidence suggests that the number of missing teeth may vary depending on the reason for seeking dental care (having symptoms versus not having symptoms” |
| Reviewer (2) | 4 | The largest limitation in this manuscript is that the fluoride aspect has not been accounted for. Toothbrushing was included in the questionnaire, but not fluoride concentrations in toothpaste, toothpaste behaviors (such as amounts of fluoride toothpaste used, rinsing with water during or after brushing), or usage of additional fluoride products. The usage of fluoride in the treatment and prevention of dental caries is of great importance and probably affects the results in this study. This is not even discussed in the manuscript, why? | We acknowledge your concern. Despite collecting information about the participant’s oral hygiene practices, certain details related to the use of fluoride in oral hygiene practices were not acquired. We acknowledge the anticaries role of fluoride and the effect of different tooth brushing behaviors on dental caries experience. Therefore, this point was addressed by including it as a new limitation to our research. | **Discussion**  **(Line 483 in Track changes file)**  **This was added as a new limitation to our research. The following statements were added**:  **“**For instance, despite collecting information about the participant’s oral hygiene practices, certain details related to the use of fluoride in these practices were not acquired. The anticaries effect of fluoride is influenced by the amount and concentration of fluoride toothpaste, and the rinsing regimen” |
| Reviewer (2) | 5 | Additionally, saliva secretion rates, and buffer capacity has not been included either which is a limitation since this affects the caries risk. | Thank you for pointing out this factor. Indeed, several studies demonstrate a relationship between dental caries and certain salivary parameters. However, dental caries is a multifactorial disease, adjusting for all confounders in one study is not feasible**[12]**. Compared to other published work**[13, 14],** our research adjusted for a large group of confounders (age, sex, BMI, medical problems, medications, dental visits, brushing and flossing frequency, consumption frequency of sugary drinks, candies, snacks, and sugary baked item, smoking status, participant’s education level, mothers’ education level, income level and the plaque index). However, we were keen to keep the reader informed of the limitations raised by reviewer (2) as to be addressed in future studies confirming/contradicting our observations. | **Discussion Section**  **(Line 495 in Track changes file)**    **Added the words** “and buffer capacity[101-104]” |
| Reviewer (2) | 6 | Regarding supplementary information, I cannot see the pictures used in the questionnaire about food preferences, and I cannot find the questionnaire used for collecting the general information. | We appreciate your feedback. We have included the POPB questionnaire per your request. We sought permission from Elsevier to reuse the questionnaire and the translated version in our future publication. The license number is 5662390874243. We did not include the questionnaire previously due to uncertainty about the terms of the permission granted to us. Further correspondence with Elsevier clearly stated that we can reuse and include the POPB questionnaire, and the Arabic version as shown in the uploaded supplementary file.  There were 4 photo-based questions (Q21-Q24). We did not include the pictures as the permission was granted to us to reuse the questionnaire in our manuscript in the format presented in the supplementary file. However, food items presented in these pictures are clearly stated in each of the photo-based questions such as (raw carrots, apples, and peanuts coated with chocolate etc). Kindly note that the pictures are available in the original questionnaire “supplementary data” and can be found using the following link: [Cross-cultural differences in lingual tactile acuity, taste sensitivity phenotypical markers, and preferred oral processing behaviors - ScienceDirect](https://www.sciencedirect.com/science/article/pii/S0950329319304653?casa_token=VmjjOUwbHmkAAAAA:e9Me7nqwOr9dVmf-XgfzaLj9ouBCCt8ToA3doKrl20OmL2RfBvUWSkHMBZSZUo1sFoBHlmQRqA) | **A supplementary data file was uploaded “S1 file”.** |
| Reviewer (3) | 1 | Would it have been meaningful to include patients with minimal missing teeth or possible none to eliminate this confounding factor? | Thank you for highlighting this point. We agree with you. Studying this in a sample of dentate individuals will improve our understanding of this relationship. It will also help us overcome the potential unwanted overestimation of the “M” component due to the different biases discussed in the manuscript. Accordingly, this point was added to the limitations of the study. | **Discussion Section**  **(Line 475 in Track changes file)**  **The following statement was added**  “To overcome such problems, future studies are encouraged to examine this potential relationship in dentate individuals.”      **(Line 528 in Track changes file)**  **The following statement was added**  “It would be crucial to investigate this potential relationship among the different mouth behavior groups in dentate individuals using the JBMB tool®” |
| Reviewer (3) | 2 | With the information you have collected would it not have been more meaningful to perform caries risk assessment? I encourage you to present it in caries risk format if you are able to. It would simplify data presentation and make comparisons with literature easier since most studies are reporting in that way. | Thank you for the suggestion. We agree with your point. However, doing this retrospectively seemed to be unfeasible since certain factors in the available caries risk assessment forms/tools were not included in our questionnaire. The ADA caries risk assessment form, Cariogram, AAPD CRA Tool, and CAries Management By Risk Assessment (CAMBRA) are an example. Nevertheless, we are planning to use the caries assessment form/tools in the near future to highlight and understand the relationship between oral processing and dental caries. | -------- |
| Reviewer (3) | 3 | What is the importance of collecting plaque indices since its relationship with dental caries has proven to be weak | We appreciate asking about this point. When conducting this study, we had the hypothesis that differences in the preferred oral processing behavior are potentially associated with differences in dental plaque levels. Based on the available evidence**[15-18]**, we assumed that differences in dental plaque levels may contribute to differences in caries experience. Accordingly, we anticipated that FPL, who preferred using their teeth during oral processing and preferred hard food textures would demonstrate lower plaque levels compared to SPL. This hypothesis was derived from a study demonstrating changes in dental plaque levels when chewing an apple**[19, 20]**. A food texture that is most likely not preferred by SPL group **[21]**.  Your feedback (comments 1 and 3) were helpful in changing the tone of our study. | **Discussion**  **(Line 419-441 in Track changes file)**  **The purpose of collecting dental plaque and its related results were**  **added and discussed per your feedback.**  (highlighted in light blue)  “In addition to assessing dental caries experience, dental plaque levels were evaluated among the different oral processing behaviors. The dental plaque (biofilm) is known for its role in the development of dental caries and periodontal disease[68, 69]. Despite being controversial[70], previous evidence indicated that certain food textures such as hard food (including apples) were found to reduce dental plaque accumulation levels[71]. Since variations in food texture preference were previously reported between the oral processing (mouth behavior) groups[7, 8, 11], it was of interest in this project to determine dental plaque levels among the different oral processing behaviors. Although plaque accumulation levels were similar between FPL and SPL, a significant difference in dental caries experience was demonstrated. This was in agreement with previous reports in which dental plaque accumulation levels demonstrated a positive but weak relationship with the dental caries experience[72-74]. The findings in this study at least partially indicate that oral processing behavior might affect an individual’s caries experience by means other than the dental plaque levels. These could be related to variations in biofilm structure and composition[69, 75, 76], or other salivary parameters such as the salivary bacterial viability[77], and salivary flow rate [52]. Indeed, preliminary data indicates a higher salivary flow rate among crunchers and suckers[52]. Although differences in salivary flow rate were observed, the sample size was small (crunchers n= 41, suckers n= 4)[52].” |

**References:**

1.	Behr D. Assessing the use of back translation: the shortcomings of back translation as a quality testing method. International Journal of Social Research Methodology. 2017;20(6):573-84. doi: 10.1080/13645579.2016.1252188.

2.	Colina S, Marrone N, Ingram M, Sánchez D. Translation Quality Assessment in Health Research: A Functionalist Alternative to Back-Translation. Evaluation & the Health Professions. 2017;40(3):267-93. doi: 10.1177/0163278716648191. PubMed PMID: 27207929.

3.	Son J. Back translation as a documentation tool. Translation & Interpreting: The International Journal of Translation and Interpreting Research. 2018;10(2):89–100.

4.	Castro ALS, Vianna MIP, Mendes CMC. Comparison of caries lesion detection methods in epidemiological surveys: CAST, ICDAS and DMF. BMC Oral Health. 2018;18(1):122. doi: 10.1186/s12903-018-0583-6.

5.	Castro ALS, Vianna MIP, Mendes CMC. The knowledge and use of population-based methods for caries detection. BMC Oral Health. 2018;18(1):153. doi: 10.1186/s12903-018-0612-5.

6.	Shen A, Bernabé E, Sabbah W. The bidirectional relationship between weight, height and dental caries among preschool children in China. PLOS ONE. 2019;14(4):e0216227. doi: 10.1371/journal.pone.0216227.

7.	Vergnes J-N, Kaminski M, Lelong N, Musset A-M, Sixou M, Nabet C, et al. Frequency and Risk Indicators of Tooth Decay among Pregnant Women in France: A Cross-Sectional Analysis. PLOS ONE. 2012;7(5):e33296. doi: 10.1371/journal.pone.0033296.

8.	Iwasaki M, Kakuta S, Ansai T. Associations among internet addiction, lifestyle behaviors, and dental caries among high school students in Southwest Japan. Sci Rep. 2022;12(1):17342. doi: 10.1038/s41598-022-22364-0.

9.	Olczak-Kowalczyk D, Krämer N, Gozdowski D, Turska-Szybka A. Developmental enamel defects and their relationship with caries in adolescents aged 18 years. Sci Rep. 2023;13(1):4932. doi: 10.1038/s41598-023-31717-2.

10.	Hawley GM, Holloway PJ, Davies RM. Dental health status associated with documented dental attendance patterns in adolescents. Community dental health. 1997;14(1):22-4. PubMed PMID: 9114545.

11.	Lopez R, Baelum V. Factors associated with dental attendance among adolescents in Santiago, Chile. BMC Oral Health. 2007;7(1):4. doi: 10.1186/1472-6831-7-4.

12.	Nascimento GG, Chaffee BW. Causal Inference in Oral Health Epidemiology. In: Peres MA, Antunes JLF, Watt RG, editors. Oral Epidemiology: A Textbook on Oral Health Conditions, Research Topics and Methods. Cham: Springer International Publishing; 2021. p. 267-78.

13.	Peng SM, Wong HM, King NM, McGrath C. Association between Dental Caries and Adiposity Status (General, Central, and Peripheral Adiposity) in 12-Year-Old Children. Caries research. 2013;48(1):32-8. doi: 10.1159/000351690.

14.	Campus G, Cagetti MG, Senna A, Blasi G, Mascolo A, Demarchi P, et al. Does Smoking Increase Risk for Caries? A Cross-Sectional Study in an Italian Military Academy. Caries research. 2011;45(1):40-6. doi: 10.1159/000322852.

15.	Laajala A, Pesonen P, Anttonen V, Laitala M-L. Association of Enamel Caries Lesions with Oral Hygiene and DMFT among Adults. Caries research. 2019;53(4):475-81. doi: 10.1159/000497358.

16.	Leroy R, Bogaerts K, Martens L, Declerck D. Risk factors for caries incidence in a cohort of Flemish preschool children. Clin Oral Investig. 2012;16(3):805-12. doi: 10.1007/s00784-011-0579-y.

17.	Warren JJ, Weber-Gasparoni K, Marshall TA, Drake DR, Dehkordi-Vakil F, Kolker JL, et al. Factors Associated with Dental Caries Experience in 1-Year-Old Children. J Public Health Dent. 2008;68(2):70-5. doi: <https://doi.org/10.1111/j.1752-7325.2007.00068.x>.

18.	Declerck D, Leroy R, Martens L, Lesaffre E, Garcia-Zattera M-J, Broucke SV, et al. Factors associated with prevalence and severity of caries experience in preschool children. Community dentistry and oral epidemiology. 2008;36(2):168-78. doi: <https://doi.org/10.1111/j.1600-0528.2007.00385.x>.

19.	Schneider HG, Knieknecht I. [Reduction of dental plaque by apples and chewing gum]. Nahrung. 1986;30(9):907-12. PubMed PMID: 3796719.

20.	Rubido S, García-Caballero L, Abeleira MT, Limeres J, García M, Diz P. Effect of chewing an apple on dental plaque removal and on salivary bacterial viability. PLOS ONE. 2018;13(7):e0199812. doi: 10.1371/journal.pone.0199812.

21.	Jeltema M, Beckley J, Vahalik J. Food texture assessment and preference based on Mouth Behavior. Food Quality and Preference. 2016;52:160-71. doi: <https://doi.org/10.1016/j.foodqual.2016.04.010>.
